# Nitrosylation Mechanisms of *Mycobacterium tuberculosis* and *Campylobacter jejuni* Truncated Hemoglobins N, O, and P

**DOI:** 10.1371/journal.pone.0102811

**Published:** 2014-07-22

**Authors:** Paolo Ascenzi, Alessandra di Masi, Grazia R. Tundo, Alessandra Pesce, Paolo Visca, Massimo Coletta

**Affiliations:** 1 Interdepartmental Laboratory of Electron Microscopy, University Roma Tre, Roma, Italy; 2 Department of Sciences, University Roma Tre, Roma, Italy; 3 Department of Clinical Sciences and Translational Medicine, University of Roma “Tor Vergata”, Roma, Italy; 4 Interuniversity Consortium for the Research on the Chemistry of Metals in Biological Systems, Bari, Italy; 5 Department of Physics, University of Genova, Genova, Italy; University of Padova, Medical School, Italy

## Abstract

Truncated hemoglobins (trHbs) are widely distributed in bacteria and plants and have been found in some unicellular eukaryotes. Phylogenetic analysis based on protein sequences shows that trHbs branch into three groups, designated N (or I), O (or II), and P (or III). Most trHbs are involved in the O_2_/NO chemistry and/or oxidation/reduction function, permitting the survival of the microorganism in the host. Here, a detailed comparative analysis of kinetics and/or thermodynamics of (*i*) ferrous *Mycobacterium tubertulosis* trHbs N and O (Mt-trHbN and Mt-trHbO, respectively), and *Campylobacter jejuni* trHb (Cj-trHbP) nitrosylation, (*ii*) nitrite-mediated nitrosylation of ferrous Mt-trHbN, Mt-trHbO, and Cj-trHbP, and (*iii*) NO-based reductive nitrosylation of ferric Mt-trHbN, Mt-trHbO, and Cj-trHbP is reported. Ferrous and ferric Mt-trHbN and Cj-trHbP display a very high reactivity towards NO; however, the conversion of nitrite to NO is facilitated primarily by ferrous Mt-trHbN. Values of kinetic and/or thermodynamic parameters reflect specific trHb structural features, such as the ligand diffusion pathways to/from the heme, the heme distal pocket structure and polarity, and the ligand stabilization mechanisms. In particular, the high reactivity of Mt-trHbN and Cj-trHbP reflects the great ligand accessibility to the heme center by two protein matrix tunnels and the E7-path, respectively, and the penta-coordination of the heme-Fe atom. In contrast, the heme-Fe atom of Mt-trHbO the ligand accessibility to the heme center of Mt-trHbO needs large conformational readjustments, thus limiting the heme-based reactivity. These results agree with different roles of Mt-trHbN, Mt-trHbO, and Cj-trHbP *in vivo*.

## Introduction

**Figure 1:**
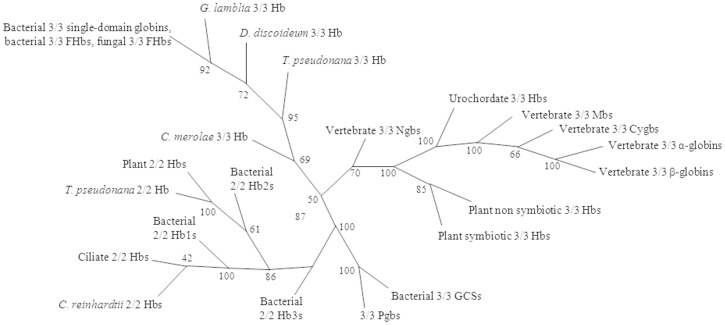
Consensus phylogenetic tree of major globin groups from the three kingdoms of life. This phylogenetic tree was based on the alignment of 150 sequences representing the following groups of globins: 10 plant non symbiotic 3/3 Hbs, 5 plant symbiotic 3/3 Hbs, 15 bacterial 3/3 globin-coupled sensors (GCSs), 4 3/3 protoglobins (Pgbs), 9 bacterial 2/2 Hb1s, 19 bacterial 2/2 Hb2s, 10 bacterial 2/2 Hb3s, 2 *Chlamydomonas reinhardtii* 2/2 Hbs, 2 ciliate 2/2 Hbs, 3 plant 2/2 Hbs, *Thalassiosira pseudonana* 2/2 Hb, 20 bacterial 3/3 flavohemoglobins (FHbs), 19 bacterial 3/3 single-domain Hbs, 9 eukaryote 3/3 FHbs, 1 diplomonad *Giardia lamblia* 3/3 Hb, and 1 mycetozoan *Dictyostelium discoideum* 3/3 Hb, *Cyanidioschyzon merolae* and *Thalassiosira pseudonana* 3/3 single-domain globins, and 3 vertebrate (*i.e.*, human, bird, and fish) 3/3 neuroglobins (Ngbs), cytoglobins (Cygbs), α- and β-globins and myoglobins (Mbs), and 2 urochordate 3/3 Hbs. Modified from [1] (Copyright (2005) National Academy of Sciences, U S A).

Based on phylogeny, the globin superfamily contains three lineages: flavohemoglobins and single domain globins (lineage 1), protoglobins (Pgb) and globin coupled sensors (lineage 2), and truncated hemoglobins (trHbs; lineage 3). Members of the globin superfamily belong to two structural classes: one showing the classical 3-on-3 α-helical sandwich (lineages 1 and 2) and one having the 2-on-2 α-helical sandwich (lineage 3) (Fig. 1). Although no definitive conclusion can be drawn about the ancestral state of the globin fold, the occurrence of the 2-on-2 fold, but not of an isolated 3-on-3 fold, in all three kingdoms of life suggests that the 2-on-2 is the ancestral fold. In an evolutionary perspective, the predominant function of globins is (pseudo-)enzymatic, with O_2_ transport and storage being specialized functions associated with the evolution of metazoans [1]–[6].

TrHbs are widely distributed in bacteria and plants and have been found in some unicellular eukaryotes. They are distantly related to the 3-on-3 globins, showing less than 20% overall identity with the latter. Phylogenetic analysis of protein sequences shows that trHbs branch into three groups, designated N (or I), O (or II), and P (or III). TrHbs belonging to groups N and O separate into two and four subgroups, respectively; trHbs belonging to group P display a level of conservation higher than those of groups N and O (Fig. 1). The overall sequence identity between trHbs from different groups is ≤20%, but may be higher than 80% within a given group. Some bacteria display multiple trHbs belonging to different groups, suggesting a scenario for the evolution of the different groups where the group O gene is the ancestor, and group N and P genes are the results of duplications and transfer events [1]–[6].

**Figure 2:**
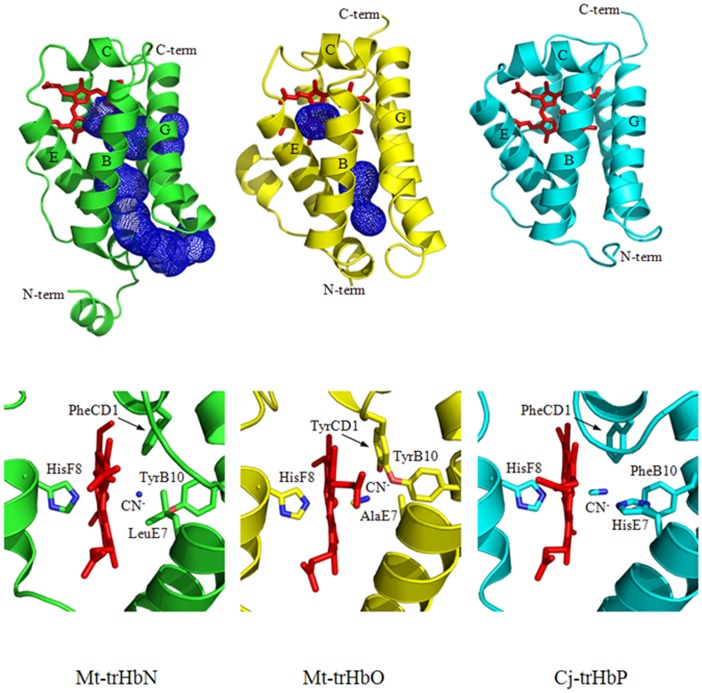
Three-dimensional structure of Mt-trHbN, Mt-trHbO, and Cj-trHbP. (Top) Ribbon views of Mt-trHbN, Mt-trHbO, and Cj-trHbP, including the heme-Fe group (red) and the protein matrix cavity/tunnel systems (blue mash). (Bottom) The heme-Fe pocket of Mt-trHbN, Mt-trHbO, and Cj-trHbP. The heme group is colored in red. The heme ligand (a cyanide ion in all the three structures) and the side chains of residues in the B10, CD1, E7 and F8 topological positions are highlighted. Atomic coordinates were taken from the PDB entries 1S56 (Mt-trHbN), 1NGK (Mt-trHbO), and 2IG3 (Cj-trHbP). All pictures have been drawn with the Swiss-PdbViewer [91].

TrHbs fold as a 2-on-2 α-helical sandwich characterized by a very short or absent A-helix, a brief CE inter-helical region, and most of the F-helix occurring as a loop, with only the B, E, G, and H α-helices surrounding the heme group. Specific residue deletions and substitutions distributed throughout the trHb sequence allow the achievement of the simplified fold, keeping at the same time a high affinity for the heme, a suitable ligand access to the heme-Fe atom, and the proper heme-Fe oxidation state. However, specific features, such as ligand entry/exit mechanisms holding to promote diffusion of ligands to/from the heme, the heme distal pocket structure and polarity, as well as ligand stabilization mechanisms distinguish members of the three trHb groups (Fig. 2) [5].

*Mycobacterium tuberculosis* (*M. tuberculosis*) possesses two trHbs, namely Mt-trHbN and Mt-trHbO, and a 3-on-3 flavohemoglobin, while *Campylobacter jejuni* (*C. jejuni*) possesses only *Campylobacter jejuni* trHb type P (Cj-trHbP) and a single-domain 3-on-3 globin [4], [5], [7]–[11].

Most trHbs are involved in the O_2_/NO chemistry and/or oxidation/reduction function, permitting the survival of the microorganism in the host [5], [11]–[13]. Noteworthy, Mt-trHbN binds reversibly isoniazid, a first-line anti-tuberculosis medication in prevention and treatment of tuberculosis, highlighting a direct role of the pro-drug to impair fundamental functions of mycobacteria, *e.g.* scavenging of reactive nitrogen and oxygen species, and metabolism [14].

Studies performed with *Mycobacterium bovis* Calmette-Guerin (BCG) demonstrated that the inactivation of the *glbN* gene impairs the ability of stationary phase cells to protect aerobic respiration from NO inhibition, suggesting that Mt-trHbN may play a vital role in protecting *M. tuberculosis* from NO toxicity *in vivo* [12], [15]. This functional assessment is supported by the observation that Mt-trHbN catalyzes the rapid oxidation of NO into nitrate [13], [16]. Heterologous expression of Mt-trHbN has also been shown to protect *Escherichia coli* against nitrosative stress [17]. On the other hand, Mt-trHbO does not detoxify NO efficiently [18], but displays peroxidase activity, suggesting an oxidation/reduction function [19]. Lastly, Cj-trHbP has been proposed to play a prominent role in *C. jejuni* respiration rather than in protection against reactive nitrogen and oxygen species. A strain of *C. jejuni* lacking trHbP turned out to be disadvantaged with respect to wild-type cells when grown under high aeration, achieving lower growth yields and consuming O_2_ at approximately half the rate displayed by wild-type cells. Although Cj-trHbP mutated cells are equally sensitive as the wild-type to NO and oxidative stress, the actual functional role of Cj-trHbP remains elusive [7], [8], [11].

Here, a detailed comparative analysis of kinetics and/or thermodynamics of (*i*) ferrous Mt-trHbN, Mt-trHbO, and Cj-trHbP (trHbN(II), Mt-trHbO(II), and Cj-trHbP(II), respectively) nitrosylation, (*ii*) nitrite-mediated nitrosylation of Mt-trHbN(II), Mt-trHbO(II), and Cj-trHbP(II), and (*iii*) NO-based reductive nitrosylation of ferric Mt-trHbN, Mt-trHbO, and Cj-trHbP (trHbN(III), Mt-trHbO(III), and Cj-trHbP(III), respectively) is reported. Ferrous and ferric Mt-trHbN and Cj-trHbP display a very high reactivity towards NO, the conversion of nitrite to NO being facilitated primarily by ferrous Mt-trHbN. This reflects the great ligand accessibility to the heme center of Mt-trHbN, and Cj-trHbP by two protein matrix tunnels and the E7-path, respectively, and the penta-coordination of the heme-Fe atom. In contrast, the accessibility to the heme center of Mt-trHbO needs large conformational readjustments, thus limiting the heme-based reactivity. These results agree with different roles of Mt-trHbN, Mt-trHbO, and Cj-trHbP *in vivo*.

## Materials

Mt-TrHbN(III), Mt-trHbO(III), and Cj-trHbP(III) were cloned, expressed and purified as previously reported [20]–[22]. Mt-TrHbN(II), Mt-trHbO(II), and Cj-trHbP(II) were obtained, under anaerobic conditions, by adding sodium dithionite (final concentration, 1.0×10^−3^ M to 5.0×10^−3^ M). The concentration of Mt-trHbN, Mt-trHbO, and Cj-trHbP stock solutions was 3.0×10^−6^ M, 4.6×10^−6^ M, and 3.2×10^−6^ M, respectively.

Gaseous NO (from Aldrich Chemical Co. (Milwaukee, WI, USA) was purified by flowing through a NaOH column in order to remove acidic nitrogen oxides. The stock NO solution was prepared anaerobically by keeping in a closed vessel the degassed 5.0×10^−3^ M phosphate buffer solution (pH = 7.0) under NO at *P* = 760.0 mm Hg (*T* = 20.0°C). The solubility of NO in the aqueous buffered solution is 2.05×10^−3^ M, at *P* = 760.0 mm Hg and *T* = 20.0°C [23]. Sodium dithionite (final concentration, 5.0×10^−4^ M) was added to NO solutions (final concentration, 1.0×10^−4^ M to 5.0×10^−4^ M).

All the other chemicals were obtained from Merck AG (Darmstadt, Germany). All products were of analytical grade and used without purification unless stated.

## Methods

### Nitrosylation of Mt-trHbN(II), Mt-trHbO(II), and Cj-trHbP(II)

Kinetics of Mt-trHbN(II), Mt-trHbO(II), and Cj-trHbP(II) nitrosylation were analyzed in the framework of the minimum reaction mechanism represented by Scheme A [23]:
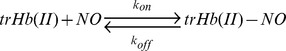
(A)


Values of the pseudo-first-order rate constant (*i.e.*, *k*) for Mt-trHbN(II), Mt-trHbO(II), and Cj-trHbP(II) nitrosylation were obtained by mixing the trHb(II) solutions (final concentration, 1.5×10^−6^ M, 2.3×10^−6^ M, and 1.6×10^−6^ M, respectively) with the NO solution (final concentration, 5.0×10^−6^ M to 1.2×10^−4^ M) under anaerobic conditions.

No gaseous phase was present. The nitrosylation of Mt-trHbN(II), Mt-trHbO(II), and Cj-trHbP(II) was monitored between 360 and 460 nm.

In view of the linear relationship between the absorbance change and the protein concentration change, values of the pseudo-first-order rate constant *k* were obtained according to Eqn. (1) [23]:
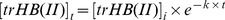
(1)


Values of the second-order rate constant for Mt-trHbN(II), Mt-trHbO(II), and Cj-trHbP(II) nitrosylation (*i.e.*, *k*_on_) were obtained from the dependence of *k* on the NO concentration (*i.e.*, [NO]), according to Eqn. (2) [23]:
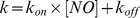
(2)where *k*_off_ is the first-order rate constant for trHb(II)-NO denitrosylation.

Values of *k*_on_ and *k*_off_ for Mt-trHbN(II)(-NO), Mt-trHbO(II)(-NO), and Cj-trHbP(II)(-NO) (de)nitrosylation were obtained at pH 7.0 and 9.0 (1.0×10^−1^ M Bis-Tris propane buffer), and 20.0°C.

### Nitrite-mediated nitrosylation of Mt-trHbN(II), Mt-trHbO(II), and Cj-trHbP(II)

Kinetics of the nitrite-mediated nitrosylation of Mt-trHbN(II), Mt-trHbO(II), and Cj-trHbP(II) were analyzed in the framework of the minimum reaction mechanism depicted in Scheme B [24]–[35]:
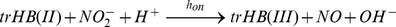
(Ba)

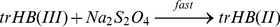
(Bb)

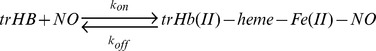
(Bc)


Values of the pseudo-first-order rate constant (*i.e.*, *h*; reaction (a) in Scheme B) for the nitrite-mediated nitrosylation of Mt-trHbN(II), Mt-trHbO(II), and Cj-trHbP(II) were determined spectrophotometrically by mixing the trHb(II) solutions (final concentration, 1.5×10^−6^ M, 2.3×10^−6^ M, and 1.6×10^−6^ M, respectively) with the nitrite solutions (final concentration, 1.0×10^−3^ M to 1.0×10^−2^ M) in the presence of sodium dithionite (final concentration, 2.0×10^−3^ M), under anaerobic conditions. Remarkably, sodium dithionite concentration lower than 1.0×10^−2^ M does not effectively reduce nitrite to NO [28]. No gaseous phase was present. The nitrite-mediated nitrosylation of Mt-trHbN(II), Mt-trHbO(II), and Cj-trHbP(II) was monitored between 360 and 460 nm.

In view of the linear relationship between the absorbance change and the protein concentration change, values of the pseudo-first-order rate constant for the nitrite-mediated nitrosylation of Mt-trHbN(II), Mt-trHbO(II), and Cj-trHbP(II) (*i.e.*, *h*) were obtained according to Eqn. (3) [24], [31], [32], [34], [35]:
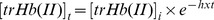
(3)


Values of the second order rate constant for the nitrite-mediated nitrosylation of Mt-trHbN(II), Mt-trHbO(II), and Cj-trHbP(II) (*i.e.*, *h*_on_) were obtained from the dependence of *h* on the nitrite concentration (*i.e.*, [NO_2_^−^]), according to Eqn. (4) [24], [31], [32], [34], [35]:
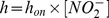
(4)


Values of *h*_on_ for the nitrite-mediated nitrosylation of Mt-trHbN(II), Mt-trHbO(II), and Cj-trHbP(II) (reaction (a) in Scheme B) were obtained between pH 6.4 and 7.8 (1.0×10^−1^ M Bis-Tris propane buffer), at 20.0°C.

### Reductive nitrosylation of Mt-trHbN(III), Mt-trHbO(III), and Cj-trHbP(III)

Kinetics and thermodynamics of the reductive nitrosylation of Mt-trHbN(III), Mt-trHbO(III), and Cj-trHbP(III) were analyzed in the framework of the minimum reaction mechanism represented by Scheme C [36]–[43]:
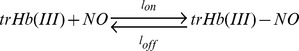
(Ca)

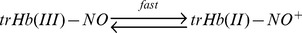
(Cb)

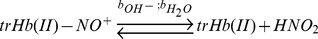
(Cc)

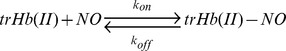
(Cd)


Values of the pseudo-first-order rate constants (*i.e.*, *l* and *b*; reactions (a) and (c) in Scheme C, respectively) and of the dissociation equilibrium constant (*i.e.*, *L* ( = *l*_off_/*l*_on_); reaction (a) in Scheme C) for Mt-trHbN(III), Mt-trHbO(III), and Cj-trHbP(III) reductive nitrosylation were obtained by mixing the trHb(III) solutions (final concentration, 1.5×10^−6^ M, 2.3×10^−6^ M, and 1.6×10^−6^ M, respectively) with the NO solution (final concentration, 2.5×10^−5^ M to 5.0×10^−4^ M) under anaerobic conditions. No gaseous phase was present. The reductive nitrosylation of Mt-trHbN(III), Mt-trHbO(III), and Cj-trHbP(III) was monitored between 360 and 460 nm.

In view of the linear relationship between the absorbance change and the protein concentration change, values of the pseudo-first-order rate constants *l* and *b* were obtained according to Eqns (5a)–(5c) [36]–[44]:
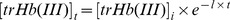
(5a)

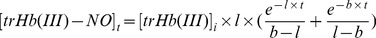
(5b)

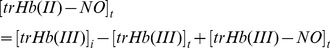
(5c)


Values of *l*_on_ and *l*_off_ (reaction (a) in Scheme C) were determined from the dependence of *l* on [NO], according to Eqn. (6) [23]:
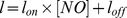
(6)


Values of *L* (reaction (a) in Scheme C) were obtained according to Eqn. (7) [23], [41], [42]:
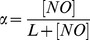
(7)


where *α* is the molar fraction of the NO-bound Mt-trHbN(III), Mt-trHbO(III), and Cj-trHbP(III). Moreover, values of *L* were estimated from the *l*_off_/*l*_on_ ratio [23].

Values of the second-order rate constant *b*_OH−_ and of the first-order rate constant *b*_H2O_ for the OH^−^- and H_2_O-catalyzed conversion of trHb(II)-NO^+^ to trHb(II), respectively (reaction (c) in Scheme C), were determined from the dependence of *b* on [OH^−^] according to Eqn. (8) [36]–[43]:
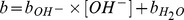
(8)


Values of *L*, *l*_on_, *l*_off_, *b*_OH−_, and *b*_H2O_ for the reductive nitrosylation of Mt-trHbN(III), Mt-trHbO(III), and Cj-trHbP(III) (reactions (3a) and (3c) in Scheme C) were obtained between pH 8.4 and pH 9.4 (1.0×10^−1^ M Bis-Tris propane buffer), at 20.0°C. Values of *k*_on_ and *k*_off_ for Mt-trHbN(II), Mt-trHbO(II), and Cj-trHbP(II) nitrosylation (Scheme A, and reaction (Cd) in Scheme C) were obtained at pH 9.0 and 20.0°C; see above).

The reductive nitrosylation of Mt-trHbN(III), Mt-trHbO(III), and Cj-trHbP(III) was also obtained anaerobically by keeping the trHb(III) solutions under purified gaseous NO (760 mmHg), between pH 8.4 and 9.4 (1.0×10^−1^ M Bis-Tris propane buffer) and 20.0°C.

### Stopped-flow apparatus

Kinetic experiments have been carried out spectrophotometrically with the BioLogic SFM 2000 (Claix, France) rapid-mixing stopped-flow apparatus at single wavelength between 360 nm and 460 nm; the dead-time of the stopped-flow apparatus was ∼1 ms and the observation chamber was 1 cm.

### Data analysis

Kinetic data obtained at different wavelengths have been normalized each other on the basis of the total absorbance change at the specific wavelength. The results are given as mean values of at least four experiments plus or minus the corresponding standard deviation. All data were analyzed using the Matlab program (The Math Works Inc., Natick, MA, USA).

## Results and Discussion

### Nitrosylation of Mt-trHbN(II), Mt-trHbO(II), and Cj-trHbP(II)

**Figure 3:**
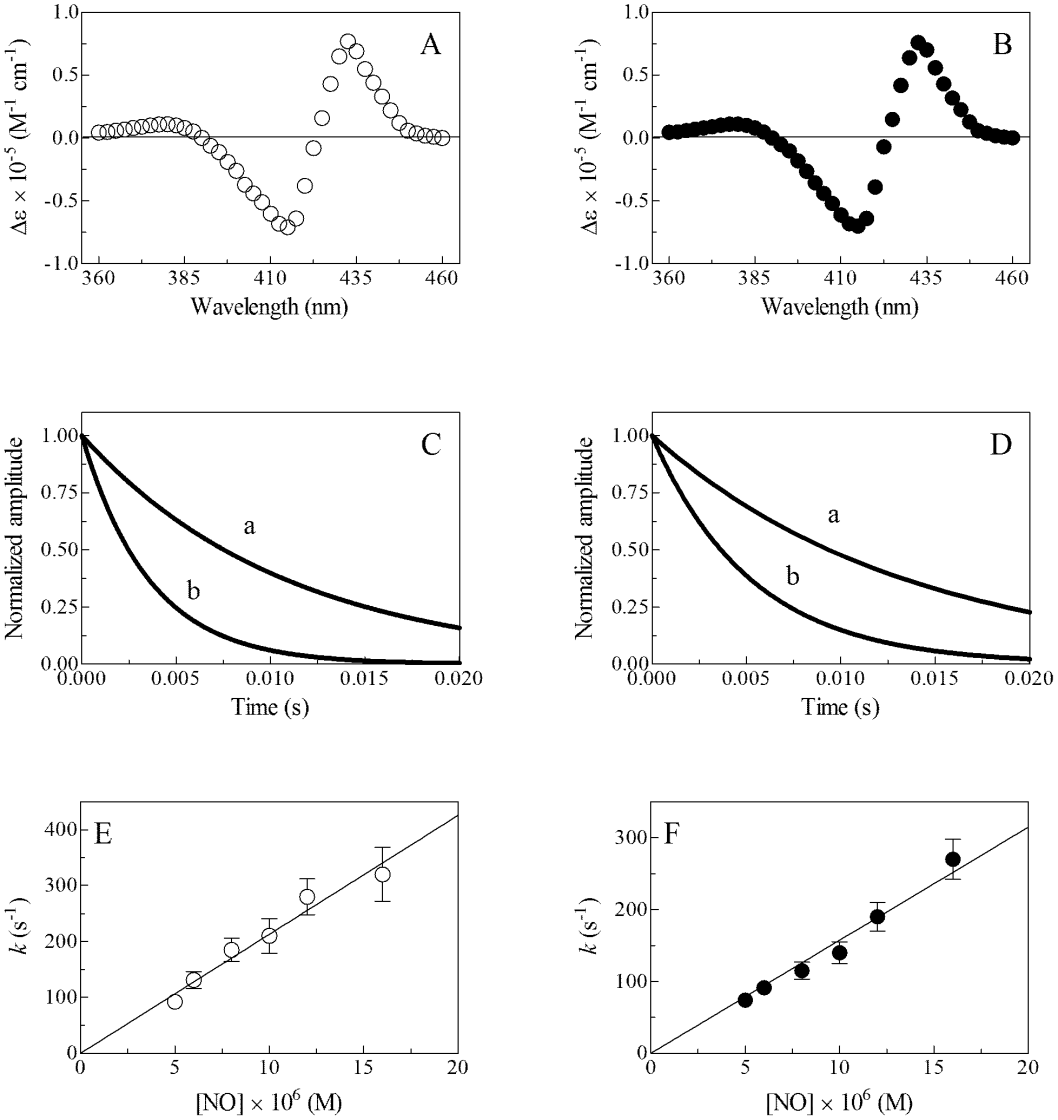
Mt-trHbN(II) nitrosylation at 20.0°C. (A) Difference absorbance spectrum of Mt-trHbN(II) *minus* Mt-trHbN(II)-NO, at pH 7.0. (B) Difference absorbance spectrum of Mt-trHbN(II) *minus* Mt-trHbN(II)-NO, at pH 9.0. (C) Normalized averaged time courses of Mt-trHbN(II) nitrosylation, at pH 7.0. The NO concentration was 5.0×10^−6^ M (trace a) and 1.2×10^−5^ M (trace b). The time course analysis according to Eqn. (1) allowed the determination of the following values of *k* = 9.2×10^1^ s^−1^ (trace a) and 2.8×10^2^ s^−1^ (trace b). (D) Normalized averaged time courses of Mt-trHbN(II) nitrosylation, at pH 9.0. The NO concentration was 5.0×10^−6^ M (trace a) and 1.2×10^−5^ M (trace b). The time course analysis according to Eqn. (1) allowed the determination of the following values of *k* = 7.4×10^1^ s^−1^ (trace a) and 1.9×10^2^ s^−1^ (trace b). (E) Dependence of the pseudo-first-order rate-constant *k* for Mt-trHbN(II) nitrosylation on the NO concentration, at pH 7.0. The analysis of data according to Eqn. (2) allowed the determination of *k*_on_ = (2.1±0.3)×10^7^ M^−1^ s^−1^. (F) Dependence of the pseudo-first-order rate-constant *k* for Mt-trHbN(II) nitrosylation on the NO concentration, at pH 9.0. The analysis of data according to Eqn. (2) allowed the determination of *k*_on_ = (1.6±0.3)×10^7^ M^−1^ s^−1^. The Mt-trHbN(II) concentration was 1.5×10^−6^ M. The NO concentration was 1.0×10^−4^ M (panels A and B). Where not shown, the standard deviation is smaller than the symbol. For details, see text.

Mixing Mt-trHbN(II), Mt-trHbO(II), and Cj-trHbP(II) with NO solutions (pH 7.0 and 9.0, 20.0°C) induces a pH-independent shift of the optical absorption maximum of the Soret band from 430–433 nm (*i.e.*, trHb(II)) to 416–418 nm (*i.e.*, trHb(II)-NO) (Fig. 3, and Figs S1 and S2 in File S1, panel A, and Table 1). pH-independent absorbance spectra of Mt-trHbN(II)-NO, Mt-trHbO(II)-NO, and Cj-trHbP(II)-NO obtained mixing Mt-trHbN(II), Mt-trHbO(II), and Cj-trHbP(II) with NO solutions correspond to those obtained by adding gaseous NO to the Mt-trHbN(II), Mt-trHbO(II), and Cj-trHbP(II) solutions. Moreover, values of λ_max_ and ε of the absorption spectra in the Soret region of Mt-trHbN(II), Mt-trHbO(II), and Cj-trHbP(II) agree with those reported in the literature [8], [20], [21], [45], [46].

**Table 1:** Values of λ_max_ and ε of the absorption spectra in the Soret region of ferric and ferrous derivatives of Mt-trHbN, Mt-trHbO, and Cj-trHbP, 20.0°C.

Derivative	λ_max_ (nm)	ε (M^−1^ cm^−1^)	pH
Mt-trHbN(III)	406	1.41×10^5^	6.4
Mt-trHbN(III)-OH^−^	410	1.25×10^5^	9.4
Mt-trHbN(III)-NO	421	1.37×10^5^	6.4 to 9.4
Mt-trHbN(II)	432	1.03×10^5^	6.4 to 9.4
Mt-trHbN(II)-NO	417	1.22×10^5^	6.4 to 9.4
Mt-trHbO(III)	409	1.04×10^5^	6.4
Mt-trHbO(III)-OH^−^	412	1.36×10^5^	9.4
Mt-trHbO(III)-NO	422	1.46×10^5^	6.4 to 9.4
Mt-trHbO(II)	430	9.20×10^4^	6.4 to 9.4
Mt-trHbO(II)-NO	416	1.28×10^5^	6.4 to 9.4
Cj-trHbP(III)	410	1.41×10^5^	6.4
Cj-trHbP(III)-OH^−^	414	1.09×10^5^	9.4
Cj-trHbP(III)-NO	420	1.28×10^5^	6.4 to 9.4
Cj-trHbP(II)	433	1.19×10^5^	6.4 to 9.4
Cj-trHbP(II)-NO	418	1.41×10^5^	6.4 to 9.4

Under all experimental conditions, the time course of NO binding to Mt-trHbN(II), Mt-trHbO(II), and Cj-trHbP(II) corresponds to a mono-molecular process for more than 80% of its course (Fig. 3, and Figs S1 and S2 in File S1, panels B and C). Values of the pseudo-first-order rate constant for Mt-trHbN(II), Mt-trHbO(II), and Cj-trHbP(II) nitrosylation (*i.e.*, *k*; Eqn. (1)) are wavelength-independent at fixed NO concentration (data not shown).

Values of *k* increase linearly with the NO concentration (Fig. 3, and Figs S1 and S2 in File S1, panels D and E). The analysis of data reported in Figure 3, and Figures S1 and S2 in File S1 (panels D and E), according to Eqn. (2), allowed the determination of values of the second-order rate constant for Mt-trHbN(II), Mt-trHbO(II), and Cj-trHbP(II) nitrosylation (*i.e.*, *k*_on_; corresponding to the slope of the linear plots), which are essentially pH-independent (Fig. 3, and Figs S1 and S2 in File S1, panels D and E). The *y* intercept of the linear plots appears very close to zero (Fig. 3, and Figs S1 and S2 in File S1, panels D and E), indicating that values of *k*_off_ for Mt-trHbN(II)-NO, Mt-trHbO(II)-NO, and Cj-trHbP(II)-NO denitrosylation are lower by at least two-orders of magnitude than values of *k* obtained at the lowest NO concentration (*i.e.*, *k*_off_<1×10^−1^ s^−1^).

Values of *k*_on_ for NO binding to Mt-trHbN(II) and Cj-trHbP(II) vary between 1.1×10^7^ M^−1^ s^−1^ and 2.1×10^7^ M^−1^ s^−1^ at pH 7.0 and pH 9.0 (Fig. 3, and Figs S1 and S2 in File S1, panels D and E, and Table 2). Values of *k*_on_ for NO binding to Mt-trHbO(II) here determined at pH 7.0 and 9.0 ( = 1.9×10^5^ M^−1^ s^−1^ and 2.3×10^5^ M^−1^ s^−1^, respectively; Fig. S1 in File S1, panels D and E, and Table 2) are in agreement with data referring of the slow nitrosylation course, reflecting 80% of the whole process, previously reported at pH 7.5 ( = 1.8×10^5^ M^−1^ s^−1^) [18]. Values of *k*_on_ for NO binding to Mt-trHbO(II) and Cj-trHbP(II) increase changing the pH from 7.0 to 9.0, whereas values of *k*_on_ for Mt-trHbN(II) nitrosylation decrease. However, the small magnitude of the pH-dependent change, essentially within the error limit, might suggest that these variations are not statistically relevant.

**Table 2:** Values of the second-order rate constant for nitrosylation of ferrous globins.

Heme-protein		*k*_on_ (M^−1^ s^−1^)
Mt-trHbN		2.1×10^7^ a
		1.6×10^7^ b
Mt-trHbO		1.9×10^5^ a
		2.3×10^5^ b
Cj-trHbP		1.1×10^7^ a
		1.7×10^7^ b
Ma-Pgb^c^		2.7×10^7^
*Arabidopsis thaliana* Hb class 1^d^		2.5×10^8^
*Glycine max* Lb^e^		1.2×10^8^
*Scapharca inaequivalvis* HbI^f^		1.6×10^7^
Horse heart Mb^g^		1.6×10^7^
Sperm whale Mb^h^		1.7×10^7^
Mouse Ngb^i^		1.5×10^8^
Tetrameric human Hb^j^	α-chains	2.6×10^7^
	β-chains	2.6×10^7^
Horse heart cytochrome *c* k		8.3
HSA-heme-Fe^l^		6.3×10^6^
Ibuprofen-HSA-heme-Fe(II)^l^		4.1×10^5^
Warfarin-HSA-heme-Fe(II)^l^		4.8×10^5^
Rabbit HPX-heme-Fe^m^		6.3×10^3^

a pH 7.0 and 20.0°C. Present study.

b pH 9.0 and 20.0°C. Present study.

c pH 7.2 and 22.0°C. From [43].

d pH 7.0 and 20.0°C. From [53].

e pH 7.0 and 20.0°C. From [49].

f pH 7.0 and 20.0°C From [50].

g pH 9.2 and 20.0°C. From [41].

h pH 7.0 and 20.0°C. From [48].

i pH 7.5 and room temperature. From [51].

j pH 7.0 and 20.0°C. From [47].

k pH 6.5 and room temperature. From [36].

l pH 7.0 and 20.0°C. From [55].

m pH 7.0 and 10.0°C. From [52].

Values of *k*_on_ for nitrosylation of ferrous heme-proteins span over eight orders of magnitude (Table 2) ([36], [41]–[43], [47]–[53] and present study), mainly reflecting the ligand accessibility to the heme distal pocket and the coordination of the heme-Fe(II) atom. Of note, H-bond interactions locking the heme distal residues TyrCD1 and TrpG8 of Mt-trHbO limits ligand access to the heme distal pocket (Fig. 2) [18]. In contrast, the low values of *k*_on_ for ferrous horse heart cytochrome *c* and rabbit hemopexin-heme-Fe (HPX-heme-Fe) nitrosylation reflect the slow rate of hexa- to penta-coordination transition of the heme-Fe(II) atom, which precedes ligand binding ([36], [52] and present study). On the other hand, the fast hexa- to penta-coordination conversion of the heme-Fe(II) atom of *Arabidopsis thaliana* hemoglobin (Hb) class 1 and *Glycine max* Lb does not affect NO binding [31], [34]. Accordingly, the decrease of the *k*_on_ value for the nitrosylation of ferrous human serum heme-Fe-albumin (HSA-heme-Fe) upon binding of warfarin and ibuprofen to the fatty acid binding site 2 (FA2) has been attributed to the allosteric drug-dependent hexa-coordination of the heme-Fe(II) atom [54]–[56].

### Nitrite-mediated conversion of Mt-trHbN(II), Mt-trHbO(II), and Cj-trHbP(II) to Mt-trHbN(II)-NO, Mt-trHbO(II)-NO, and Cj-trHbP(II)-NO

**Figure 4:**
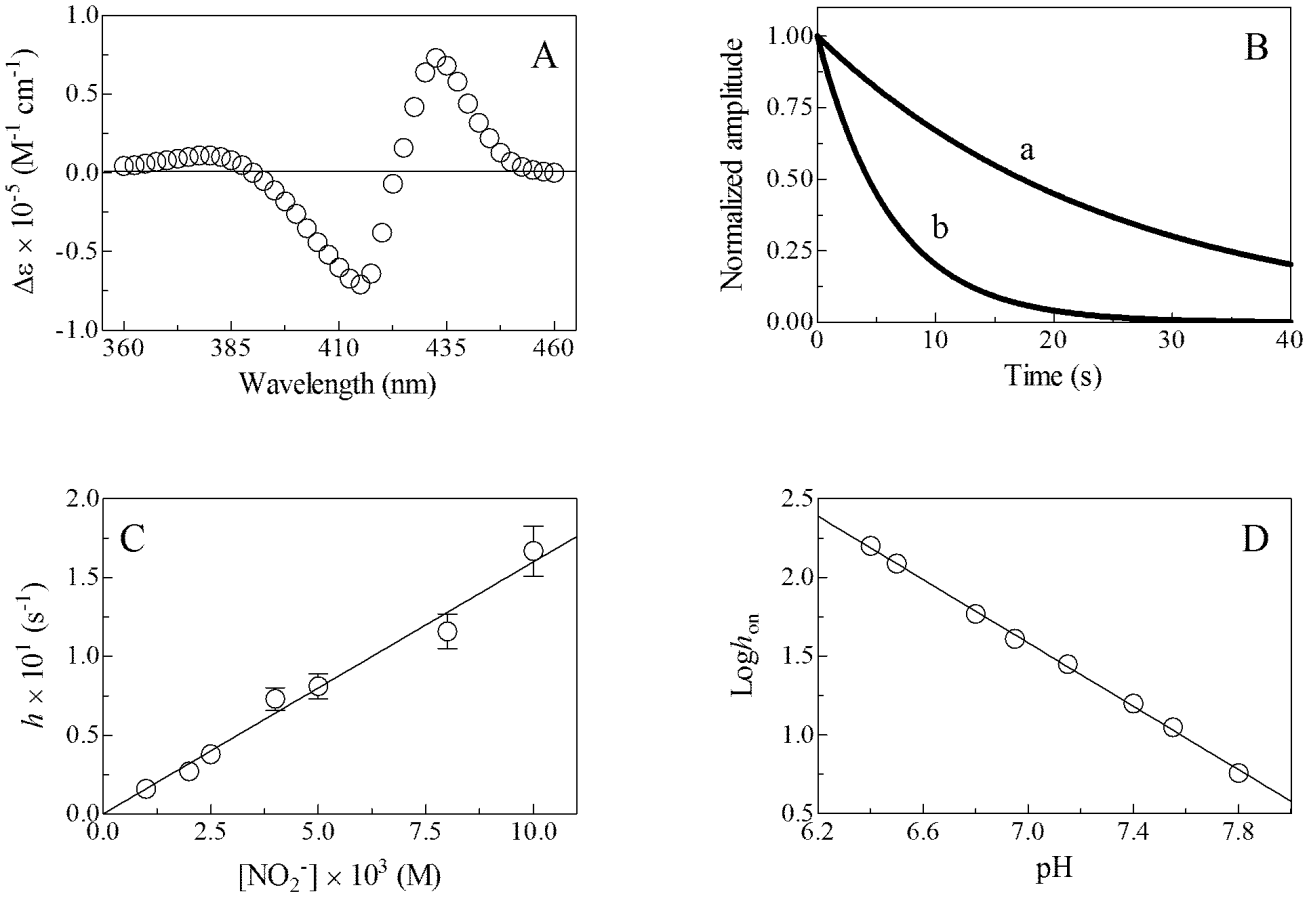
Nitrite-mediated nitrosylation of Mt-trHbN(II), at 20.0°C. (A) Difference absorbance spectrum of Mt-trHbN(II) *minus* Mt-trHbN(II)-NO, at pH 7.4. (B) Normalized averaged time courses of nitrite-mediated nitrosylation of Mt-trHbN(II), at pH 7.4. The nitrite concentration was 2.5×10^−3^ M (trace a) and 1.0×10^−2^ M (trace b). The time course analysis according to Eqn. (3) allowed the determination of the following values of *h* = 4.0×10^−2^ s^−1^ (trace a) and 1.6×10^−1^ s^−1^ (trace b). (C) Dependence of *h* on [NO_2_^−^] for nitrite-mediated nitrosylation of Mt-trHbN(II), at pH 7.4. The continuous line was generated from Eqn. (4) with *h*_on_ = (1.6±0.2)×10^1^ M^−1^ s^−1^. (D) pH-Dependence of *h*_on_ for the nitrite-mediated nitrosylation of Mt-trHbN(II). The slope of the continuous line was −1.00±0.01. The Mt-trHbN(II) concentration was 1.5×10^−6^ M. Where not shown, standard deviation is smaller than the symbol. For details, see text.

Mixing Mt-trHbN(II), Mt-trHbO(II), and Cj-trHbP(II) with nitrite solutions (between pH 6.4 and 7.8, 20.0°C) induces a pH-independent shift of the optical absorption maximum of the Soret band from 430–433 nm (*i.e.*, trHb(II)) to 416–418 nm (*i.e.*, trHb(II)-NO) (Fig. 4, and Figs S3 and S4 in File S1, panel A, and Table 1). pH-independent absorbance spectra of Mt-trHbN(II)-NO, Mt-trHbO(II)-NO, and Cj-trHbP(II)-NO obtained mixing Mt-trHbN(II), Mt-trHbO(II), and Cj-trHbP(II) with nitrite solutions correspond to those obtained by adding either NO solutions or gaseous NO to the Mt-trHbN(II), Mt-trHbO(II), and Cj-trHbP(II) solutions. Moreover, values of λ_max_ and ε of the absorption spectra in the Soret region of Mt-trHbN(II), Mt-trHbO(II), and Cj-trHbP(II) derivatives agree with those reported in the literature [8], [20], [21], [45], [46].

Under all experimental conditions, the time course of the nitrite-mediated conversion of Mt-trHbN(II), Mt-trHbO(II), and Cj-trHbP(II) to Mt-trHbN(II)-NO, Mt-trHbO(II)-NO, and Cj-trHbP(II)-NO, respectively, corresponds to a mono-molecular process for more than 90% of its course (Fig. 4, and Figs S3 and S4 in File S1, panel B). Values of the pseudo-first-order rate constant for the nitrite-mediated conversion of Mt-trHbN(II), Mt-trHbO(II), and Cj-trHbP(II) to Mt-trHbN(II)-NO, Mt-trHbO(II)-NO, and Cj-trHbP(II)-NO, respectively, (*i.e.*, *h*; Eqn. (3)) are wavelength-independent at fixed nitrite concentration (data not shown). In agreement with the literature [28], values of *h* are independent of the dithionite concentration ranging between 1.0×10^−3^ M and 5.0×10^−3^ M (data not shown).

Values of *h* increase linearly with the nitrite concentration (Fig. 4, and Figs S3 and S4 in File S1, panel C). The analysis of data reported in Figure 4, and Figure S3 and S4 in File S1 (panel C) according to Eqn. (4) allowed the determination of values of the second-order rate constant for the nitrite-mediated conversion of Mt-trHbN(II), Mt-trHbO(II), and Cj-trHbP(II) to Mt-trHbN(II)-NO, Mt-trHbO(II)-NO, and Cj-trHbP(II)-NO, respectively, (*i.e.*, *h*_on_; corresponding to the slope of the linear plots). The *y* intercept of the linear plots corresponds to zero, indicating that the nitrite-mediated conversion of Mt-trHbN(II), Mt-trHbO(II), and Cj-trHbP(II) to Mt-trHbN(II)-NO, Mt-trHbO(II)-NO, and Cj-trHbP(II)-NO, respectively, can be considered as an irreversible process.

Values of the rate constants for the nitrite-mediated conversion of Mt-trHbN(II), Mt-trHbO(II), and Cj-trHbP(II) to Mt-trHbN(II)-NO, Mt-trHbO(II)-NO, and Cj-trHbP(II)-NO, respectively, are lower by several orders of magnitude than those of trHb(II) nitrosylation (Tables 1 and 2). This indicates that the formation of the transient Mt-trHbN(III), Mt-trHbO(III), and Cj-trHbP(III) species (Scheme B), which are quickly converted to Mt-trHbN(II), Mt-trHbO(II), and Cj-trHbP(II), respectively, by reaction with sodium dithionite, represents the rate-limiting step of the nitrite-mediated conversion of trHb(II) to trHb(II)-NO.

As reported for most heme-proteins [24]–[32], [34], [35], the nitrite-mediated conversion of Mt-trHbN(III), Mt-trHbO(II), and Cj-trHbP(II) to Mt-trHbN(II)-NO, Mt-trHbO(II)-NO, and Cj-trHbP(II)-NO, respectively, requires one proton for the NO and OH^−^ formation (Scheme B). Indeed, on increasing the proton concentration by one pH unit, the rate of the nitrite-mediated conversion of trHbN(II) to trHbP(II)-NO (*i.e.*, Log *h*_on_) increases by one-order of magnitude (Fig. 4, and Figs S3 and S4 in File S1, panel D). However, the increase of *h*_on_ for the nitrite-mediated conversion of human cytoglobin on pH decrease has been interpreted accounting for the reversible pH-dependent penta-to-hexa-coordination transition of the heme-Fe(II) atom [33].

Values of *h*_on_ for nitrite-mediated conversion of ferrous heme-proteins to their ferrous nitrosylated derivatives range between 7.0×10^−2^ M^−1^ s^−1^ and 6.8×10^1^ M^−1^ s^−1^ (Table 3) ([25], [26], [29], [31]–[35] and present study), reflecting the different structural and chemical features of the heme site [5], [57]–[67]. Values of *h*_on_ for NO_2_^−^ binding to penta-coordinated ferrous Mt-trHbN, Cj-trHbP, carp myoglobin (Mb)-1, carp Mb-2, horse heart Mb, and sperm whale Mb, ranging between 1.8 M^−1^ s^−1^ and 1.6×10^1^ M^−1^ s^−1^, are among the fastest observed (Table 3). Of note, the low reactivity of Mt-trHbO reflects the unfavorable ligand accessibility to the heme pocket due to the locked conformation(s) of the heme distal residues TyrCD1 and TrpG8 (Fig. 2) [18]. The very different values of the interconversion rate for the hexa- to penta-coordination of the heme-Fe(II) atom modulates kinetics of nitrite binding to *Synechocystis* Hb, *Arabidopsis thaliana* nonsymbiotic Hbs classes 1 and 2, and rice nonsymbiotic Hb class 1, human Cygb, and horse heart cytochrome *c* [18], [31], [33], [34]. Accordingly, changes of the *h*_on_ values for the nitrite-mediated conversion of ferrous human neuroglobin (Ngb) to its ferrous nitrosylated derivative (from 1.2×10^−2^ M^−1^ s^−1^ to 1.2×10^−1^ M^−1^ s^−1^) reflect the reversible redox-linked hexa-to-penta-coordination transition of the heme-Fe(II) atom. In fact, under oxidative conditions, the formation of the Cys46-Cys55 bridge stabilizes the high-reactive penta-coordinated heme-Fe(II) atom, thus facilitating the reaction. In contrast, under reductive conditions, the cleavage of the Cys46-Cys55 bridge leads to the formation of the low-reactive hexa-coordinated heme-Fe(II) atom [32]. Similarly, the inhibition of nitrite-dependent conversion of ferrous HSA-heme-Fe to the nitrosylated derivative reflects warfarin binding to the FA2 site with the concomitant penta-to-hexa coordination interconversion of the heme-Fe(II) atom [35], Lastly, the nitrite-mediated conversion of ferrous human Hb to the nitrosylated derivative is modulated allosterically, inositol hexakisphosphate impairing the reactivity of ferrous human Hb stabilizing the low-reactivity T-state [25], [26].

**Table 3:** Values of the second-order rate constant for the nitrite-mediated nitrosylation of ferrous globins.

Heme-protein		*h*_on_ (M^−1^ s^−1^)
Mt-trHbN^a^		1.6×10^1^
Mt-trHbO^a^		3.8×10^−1^
Cj-trHbP^a^		4.3
*Synechocystis* Hb^b^		6.8×10^1^
*Arabidopsis thaliana* Hb class 1^c^		2.0×10^1^
*Arabidopsis thaliana* Hb class 2^c^		4.9
Rice non symbiotic Hb class 1^b^		3.3×10^1^
Carp Mb-1^d^		5.3
Carp Mb-2^d^		1.8
Horse heart Mb^e^		2.9
Sperm whale Mb^f^		6.0
	HisE7Ala^e^	1.8
	HisE7Leu^e^	<0.2
Mouse Ngb^g^		5.1
Human Cygb^h^		1.4×10^−1^
Human Ngb	Cys46-Cys55^e^^,^ i	1.2×10^−1^
	Cys46/Cys55^e^^,^ j	1.2×10^−2^
Tetrameric human Hb	T state^f^	1.2×10^−1^
	R state^f^	6.0
Horse heart cytochrome *c* k		7.0×10^−2^
HSA-heme-Fe^l^		1.3
Warfarin-HSA-heme-Fe(II)^l^		9.3×10^−2^

a pH 7.4 and 20.0°C. Present study.

b pH 7.0; unknown temperature [31].

c pH 7.4 and 25.0°C. From [34].

d pH 7.6 and 25.0°C. From [90].

e pH 7.4 and 25.0°C. From [32].

f pH 7.4 and 25.0°C. From [25].

g pH 7.4 and 25.0°C. From [29].

h pH 7.0 and 25.0°C. From [33].

i In “Human Ngb Cys46–Cys55”, the Cys46 and Cys55 residues form an intramolecular disulphide bond.

j In “Human Ngb Cys46/Cys55”, the Cys46 and Cys55 residues do not form the intramolecular disulphide bond.

k pH 7.4 and 25.0°C. From [33].

l pH 7.4 and 20.0°C. From [35].

### Reductive nitrosylation of Mt-trHbN(III), Mt-trHbO(III), and Cj-trHbP(III)

The reductive nitrosylation of Mt-trHbN(III), Mt-trHbO(III), and Cj-trHbP(III) occurs only at pH≥8.4. Of note, Mt-trHbN(III) binds reversibly NO without converting to Mt-trHbN(II)-NO at pH 7.5 [68].

**Figure 5:**
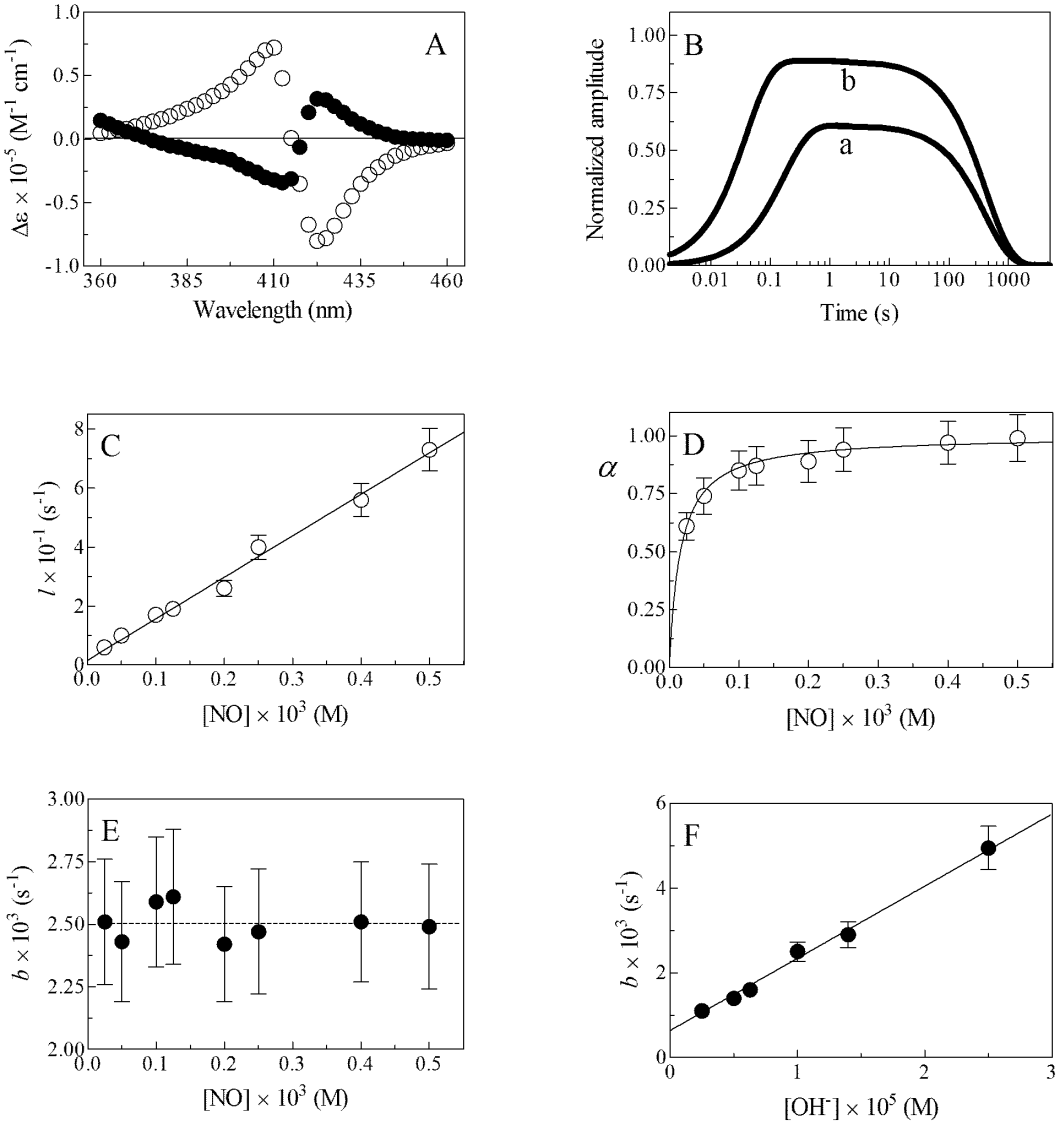
Reductive nitrosylation of Mt-trHbN(III), at 20.0°C. (A) Difference absorbance spectra of Mt-trHbN(III) *minus* Mt-trHbN(III)-NO and of Mt-trHbN(III)-NO *minus* Mt-trHbN(II)-NO (open and fillrd circles, respectively), at pH 9.0. (B) Normalized averaged time courses of Mt-trHbN(III) reductive nitrosylation, at pH 9.0. The NO concentration was 2.5×10^−5^ M (trace a) and 2.0×10^−4^ M (trace b). The time course analysis according to Eqns (5a)–(5c) allowed the determination of the following values of parameters α, *l*, and *b*: trace a - α = 0.61, *l* = 6.0 s^−1^, and *b* = 2.5×10^−3^ s^−1^; and trace b - α = 0.89, *l* = 2.6×10^1^ s^−1^, and *b* = 2.4×10^−3^ s^−1^. (C) Dependence of *l* on [NO] for Mt-trHbN(III) reductive nitrosylation, at pH 9.0. The continuous line was generated from Eqn. (6) with *l*_on_ = (1.4±0.2)×10^5^ M^−1^ s^−1^ and *l*_off_ = 1.6±0.2 s^−1^. (D) Dependence of α on [NO] for Mt-trHbN(III) reductive nitrosylation, at pH 9.0. The continuous line was generated from Eqn. (7) with *L* = (1.6±0.2)×10^−5^ M. (E) Dependence of *b* on [NO] for Mt-trHbN(III) reductive nitrosylation, at pH 9.0. The average *b* value is 2.5×10^−3^ s^−1^ (dashed line). (F) Dependence of *b* on [OH^−^] for Mt-trHbN(III) reductive nitrosylation. The continuous line was generated from Eqn. (8) with *b*_OH−_ = (1.7±0.2)×10^2^ M^−1^ s^−1^ and *b*_H2O_ = (6.4±0.7)×10^−4^ s^−1^. The Mt-trHbN(III) concentration was 1.5×10^−6^ M. Where not shown, standard deviation is smaller than the symbol. For details, see text.

Mixing Mt-trHbN(III), Mt-trHbO(III), and Cj-trHbP(III) with NO solutions (between pH 8.4 and 9.4, 20.0°C) induces a shift of the optical absorption maximum of the Soret band from 406–414 nm (*i.e.*, trHb(III)) to 420–422 nm (*i.e.*, trHb(III)-NO) (Fig. 5, and Figs S5 and S6 in File S1, panel A, and Table 1). Then, the optical absorption maximum of the Soret band shifts from 420–422 nm (*i.e.*, trHb(III)-NO) to 416–418 nm (*i.e.*, trHb(II)-NO) (Fig. 5, and Figs S5 and S6 in File S1, panel A, and Table 1). Absorbance spectra of Mt-trHbN(II)-NO, Mt-trHbO(II)-NO, and Cj-trHbP(II)-NO obtained mixing Mt-trHbN(III), Mt-trHbO(III), and Cj-trHbP(III) with NO solutions (Fig. 5, and Figs S5 and S6 in File S1, panel A, and Table 1) correspond to those obtained by: (*i*) adding gaseous NO to the Mt-trHbN(II), Mt-trHbO(II), and Cj-trHbP(II) solutions (Fig. 3, and Figs S1 and S2 in File S1, panel A, and Table 1), and (*ii*) mixing Mt-trHbN(II), Mt-trHbO(II), and Cj-trHbP(II) with nitrite solutions in the presence of dithionite (Fig. 4, and Figs S3 and S4 in File S1, panel A, and Table 1). Moreover, values of λ_max_ and ε of the absorption spectra in the Soret region of Mt-trHbN, Mt-trHbO, and Cj-trHbP derivatives agree with those reported in the literature [8], [20], [21], [45], [46], [68]–[70].

Over the whole NO concentration range explored, the time course for Mt-trHbN(III), Mt-trHbO(III), and Cj-trHbP(III) reductive nitrosylation corresponds to a biphasic process (Fig. 5, and Figs S5 and S6 in File S1, panel B); values of *l* and *b* are wavelength-independent at fixed NO concentration (data not shown).

The first step of kinetics for Mt-trHbN(III), Mt-trHbO(III), and Cj-trHbP(III) reductive nitrosylation (indicated by *l* in Scheme C) is a bimolecular process as observed under pseudo-first order conditions (Fig. 5, and Figs S5 and S6 in File S1, panel C) at all the pH values investigated. Plots of *l* versus [NO] are linear (Eqn. (6)), the slope corresponding to *l*_on_. Values of the second-order rate constant *l*_on_ over the pH range explored (*i.e.*, from pH 8.4 to 9.4; Tables S1–S3 in File S1) vary between 1.1×10^5^ M^−1^ s^−1^ and 2.3×10^5^ M^−1^ s^−1^ (for Mt-trHbN(III)), between 8.5×10^3^ M^−1^ s^−1^ and 2.1×10^4^ M^−1^ s^−1^ (for Mt-trHbO(III)), and between 8.9×10^4^ M^−1^ s^−1^ and 1.7×10^5^ M^−1^ s^−1^ (for Cj-trHbP(III)). The *y* intercept of plots of *l* versus [NO] corresponds to the dissociation rate constant *l*_off_ (Eqn. (6)), whose values over the pH range explored (*i.e.*, from pH 8.4 to 9.4; Tables S1–S3 in File S1) vary between 1.5 s^−1^ and 2.3 s^−1^ (for Mt-trHbN(III)), between 1.3 s^−1^ and 2.4 s^−1^ (for Mt-trHbO(III)), and between 5.9 s^−1^ and 8.3 s^−1^ (for Cj-trHbP(III)).

Between pH 8.4 and pH 9.4, the molar fraction of trHb(III)-NO (*i.e.*, α, Eqn. (7)) increases as a function of free [NO], tending to level off at [NO] >10×*L*; the analysis of data according to Eqn. (7) allowed to calculate values of *L* (Fig. 5, and Figs S5 and S6 in File S1, panel D). Values of *L* over the pH range explored (*i.e.*, from pH 8.4 to 9.4; Tables S1–S3 in File S1) vary between 9.3×10^−6^ M and 1.9×10^−5^ M (for Mt-trHbN(III)), between 9.8×10^−5^ M and 2.1×10^−4^ M (for Mt-trHbO(III)), and between 3.1×10^−5^ M and 7.9×10^−5^ M (for Cj-trHbP(III)). According to the trHb(III):NO 1∶1 stoichiometry of reaction Ca in Scheme C, the Hill coefficient *n* is 1.00±0.02. As expected for a simple system [23], values of *L* correspond to those of *l*_off_/*l*_on_, under all experimental conditions (Table 4 and Tables S1–S3 in File S1).

**Table 4:** Values of kinetic and thermodynamic parameters for reductive nitrosylation of ferric heme-proteins.

Heme-protein		*l*_on_ (M^−1^ s^−1^)	*l*_off_ (s^−1^)	*L* (M)	*l*_off_/*l*_on_ (M)	*b*_OH−_ (M^−1^ s^−1^)	*b*_H2O_ (s^−1^)
Mt-trHbN		1.4×10^5^ a	1.6^a^	1.6×10^−5^ a	1.1×10^−5^ a	1.7×10^2^ b	6.4×10^−4^ b
Mt-trHbO		9.2×10^3^ a	2.1^a^	1.9×10^−4^ a	2.3×10^−4^ a	2.4×10^2^ b	2.9×10^−4^ b
Cj-trHbP		1.1×10^5^ a	8.1^a^	6.5×10^−5^ a	7.4×10^−5^ a	9.1×10^2^ b	4.8×10^−4^ b
Ma-Pgb		4.8×10^4^ c	2.6^c^	6.1×10^−5^ c	5.4×10^−5^ c	2.9×10^3^ d	4.1×10^−4^ d
*Glycine max* Lb^e^		1.4×10^5^ e	3.0^e^	2.1×10^−5^ e	2.1×10^−5^ e	3.3×10^3^ f	3.0×10^−4^ f
*Scapharca inaequivalvis* HbI		3.2×10^1^ g	<1×10^−3^ g	n.d.	3.1×10^−5^ g	>2×10^6^ h	n.d.
Horse heart Mb		6.8×10^4^ i	5.2^i^	1.2×10^−4^ i	7.6×10^−5^ i	3.9×10^2^ j	n.d
Sperm whale Mb		1.9×10^5^ k	1.4×10^l^	7.7×10^−5^ k	n.d.	3.2×10^2^ m	n.d.
Human Ngb	Cys46–Cys55^n^	2.1×10^1^ o	2.5×10^−3^ o	n.d.	1.2×10^−4^ p	≥2×10^6^ q	n.d.
	Cys46/Cys55^r^	2.9^o^	2.0×10^−3^ o	n.d.	1.9×10^−4^ p	≥5×10^5^ q	n.d.
Tetrameric human Hb		n.d.	n.d.	8.3×10^−5^ s	n.d.	3.2×10^3^ t	1.1×10^−3t^
Horse cytochrome *c*		n.d.	n.d.	7.1×10^−5^ u	n.d.	1.5×10^3^ m	n.d.
HSA-heme-Fe		2.1×10^4^ v	3.1×10^−1^ v	1.8×10^−5^ v	1.5×10^−5^ v	4.4×10^3^ w	3.5×10^−4^ w
Rabbit HPX-heme-Fe		1.3×10^1^ x	≤10^−4^ x	n.d.	≤8×10^−6^ x	≥7×10^5^ y	n.d.

a pH 9.0 and 20.0°C. Present study.

b 20.0°C. Present study.

c pH 7.2 and 22.0°C. From [43].

d 22.0°C. From [43].

e pH 7.3 and 20.0°C. From [39].

f 20.0°C. From [39].

g pH 7.5 and 20.0°C. From [38].

h Derived from values of *b* and [OH^−^] [38], at 20.0°C.

i pH 9.2 and 20.0°C. From [41].

j Derived from values of *b* and [OH^−^] [41], at 20.0°C.

k pH 8.79 and room temperature. From [37].

l Derived from values of *l*_on_ and *L* [37], at pH 8.79 and room temperature.

m Room temperature. From [37].

n In “Human Ngb Cys46–Cys55”, the Cys46 and Cys55 residues form an intramolecular disulphide bond.

o pH 7.0 and room temperature. From [74].

p Derived from values of *l*_on_ and *l*_off_ [74], at room temperature.

q Derived from values of *b* and [OH^−^] [74], at pH 7.0 and room temperature.

r In “Human Ngb Cys46/Cys55”, the Cys46 and Cys55 residues do not form the intramolecular disulphide bond.

s pH 7.1 and room temperature. From [37].

t Room temperature. From [37].

u pH 8.35 and room temperature. From [37].

v pH 7.5 and 20.0°C. From [42].

w 20.0°C. From [42].

x pH 7.0 and 10.0°C. From [40].

y 10.0°C. From [40].

n.d., not determined.

The second step of kinetics for Mt-trHbN(III), Mt-trHbO(III), and Cj-trHbP(III) reductive nitrosylation (indicated by Cb-Cd in Scheme C) follows a [NO]-independent monomolecular behavior (Fig. 5, and Figs S5 and S6 in File S1, panel E) at all the pH values investigated. According to Scheme C, the value of *b* increases linearly on increasing [OH^−^] (*i.e.*, from pH 8.4 to 9.4; Fig. 5, and Figs S5 and S6 in File S1, panel F, and Tables S1–S3 in File S1). The slope and the *y* intercept of the plot of *b* versus [OH^−^] correspond to *b*_OH−_ and *b*_H2O_ values. Values of *b*_OH−_ and *b*_H2O_ for for Mt-trHbN(III), Mt-trHbO(III), and Cj-trHbP(III) reductive nitrosylation are 1.7×10^2^ M^−1^ s^−1^ and 6.4×10^−4^ s^−1^, 2.4×10^2^ M^−1^ s^−1^ and 2.9×10^−4^ s^−1^, and 9.1×10^2^ M^−1^ s^−1^ and 4.8×10^−4^ s^−1^, respectively (Table 4).

Over the whole pH range explored (*i.e.*, from pH 8.4 to 9.4), the reductive nitrosylation of Mt-trHbN(III), Mt-trHbO(III), and Cj-trHbP(III) is an irreversible process, as the spectra of Mt-trHbN(II)-NO, Mt-trHbO(II)-NO, and Cj-trHbP(II)-NO revert to Mt-trHbN(II), Mt-trHbO(II), and Cj-trHbP(II) instead of Mt-trHbN(III), Mt-trHbO(III), and Cj-trHbP(III) by merely pumping off gaseous NO or bubbling helium through the Mt-trHbN(II)-NO, Mt-trHbO(II)-NO, and Cj-trHbP(II)-NO solutions; however, the denitrosylation process (*i.e.*, the Mt-trHbN(II), Mt-trHbO(II), and Cj-trHbP(II) formation) needs about 12 hours to be completed.

Under all experimental conditions, free Mt-trHbN(II), Mt-trHbO(II), and Cj-trHbP(II) was never detected spectrophotometrically because of the very rapid reaction of Mt-trHbN(II), Mt-trHbO(II), and Cj-trHbP(II) with NO (*k*_on_≥8.6×10^5^ M^−1^ s^−1^; Table 1).

Ferric Mt-trHbN, Cj-trHbP, *Methanosarcina acetivorans* protoglobin (Ma-Pgb), horse heart Mb, sperm whale Mb, and HSA-heme-Fe react rapidly with NO ([41], [43], [48], [55] and present study), even though the binding of a weak exogenous ligand (*i.e.*, a water molecule) occurs likely at the sixth coordination position of the heme-Fe(III) atom [5], [57], [58], [66], [67], [71]. As reported above, the low reactivity of Mt-trHbO reflects the unfavorable ligand accessibility to the heme distal pocket [18]. Unexpectedly, the very low value of *l*_on_ for the reductive nitrosylation of penta-coordinated *Scapharca inaequivalvis* HbI may reflect either the non-occurrence of the heme-Fe(III)-NO intermediate or the concomitant nitrosation of the Cys92 residue affecting the heme-Fe reactivity [38]. On the other hand, the reactivity of ferric hexa-coordinated *Glycine max* Lb, horse cytochrome *c*, and rabbit HPX-heme-Fe ([37], [39], [40], [72] and present study) reflects the hexa- to penta-coordination conversion of the heme-Fe atom ([59], [65], [73] and present study) (Table 4). As reported for the nitrite-mediated nitrosylation of ferrous human Ngb [32] (Table 3), NO binding to ferric human Ngb is modulated by the formation and cleavage of the Cys46-Cys55 disulphide bridge [74].

Values of *l*_off_ for NO dissociation from nitrosylated heme-Fe(III) proteins span over five orders of magnitude (Table 4) ([37]–[43], [74] and present study), reflecting the different stabilization mode of the heme-Fe bound ligand by heme distal residues [5], [54], [58], [59], [66], [71], [73], [75]–[79].

Although values of *l*_on_ and *l*_off_ NO binding to heme-Fe(III) proteins are very different (Table 4) ([37]–[43], [74] and present study), values of *L* ( = *l*_off_/*l*_on_) are similar (Table 4) ([37]–[43], [74] and present study), indicating the occurrence of kinetic compensation phenomena.

Values of *b*_OH−_ for reductive nitrosylation of heme-Fe(III) proteins, spanning over four orders of magnitude (Table 4) ([37]–[43], [74] and present study), possibly reflect either the different OH^−^ accessibility to the heme pocket ([5], [23], [58]–[60], [63], [64], [67], [71], [75], [80]–[84]) or the heme-Fe(III) protein reduction potentials [37], [39]. Although the values of *b*_OH−_ and *b*_H2O_ cannot be compared directly, OH^−^ ions catalyze reductive nitrosylation of heme-Fe(II)-NO^+^ much more efficiently than H_2_O. Moreover, the linear dependence of *b* on [OH^−^] indicates that no additional elements appear to be involved in irreversible reductive nitrosylation of Mt-trHbN, Mt-trHbO, and Cj-trHbP (present study), as previously reported for related heme-Fe(III) proteins (reaction (c) in Scheme C; Fig. 5, and Figs S5 and S6 in File S1, panel E) [37]–[43], [74].

Reductive nitrosylation of most ferric heme-proteins is limited by the OH^−^-mediated reduction of the heme-Fe(II)-NO^+^ complex to heme-Fe(II) (reaction (c) in Scheme C) [37]–[39], [41]–[43], [74]. By contrast, NO binding to hexa-coordinated horse cytochrome *c* and ferric rabbit HPX-heme (reaction (a) in Scheme C) represents the rate-limiting step [37], [40] (Tables 2 and 4 for comparison).

## Conclusion and Perspectives

The occurrence of different types of trHbs (*i.e.*, trHbN, trHbO and trHbP) in bacteria, plants and some unicellular eukaryotes (Fig. 1) opens the question of their role, also in view of their frequently simultaneous presence in the same organism; this envisages the possibility that different types of trHbs reflect different physiological roles in these organisms [1]–[6], [11].

The high reactivity of Mt-trHbN and Cj-trHbP reflects both the penta-coordination of the heme-Fe-atom and the ligand accessibility to the heme pocket (Fig. 2). Indeed, the ligand access pathway through protein matrix tunnels in penta-coordinated Mt-trHbN (Fig. 2) [5], [83] and the E7-path in penta-coordinated Cj-trHbP (Fig. 2) [5], [22], [66] is characterized by the lowest energy barrier, with values close to those displayed by penta-coordinated sperm whale Mb. Of note, ligand entry to and exit from the heme distal pocket of sperm whale Mb is modulated by gating movement of the HisE7 residue [75]. In contrast, the low reactivity of penta-coordinated Mt-trHbO [21] reflects H-bond interactions that lock the heme distal residues TyrCD1 and TrpG8 into a conformation(s) that limits ligand access to the heme distal pocket (Fig. 2) [18], [70]. Of note, the E7-path appears to sustain ligand diffusion to the heme distal cavity of Mt-trHbO; indeed, Mt-trHbO hosts two protein matrix cavities instead of the protein matrix tunnel occurring in Mt-trHbN (Fig. 2) [84].

*M. tuberculosis* is massively exposed to NO during its intra-macrophagic life, and Mt-trHbN has been shown to play a prominent role in protection from nitrosative stress [16]. Therefore, Mt-trHbN is most likely to function as NO dioxygenase that converts toxic NO into harmless nitrate in the presence of oxygen, and relieves toxicity due to NO and nitrosative stress. This is also consistent with the NO-inducible response of the gene encoding for trHbN in *M. tuberculosis*, both *in vitro* and inside infected macrophages [85]. Of note, also the promoter of the trHbO gene is induced during macrophage infection, though it poorly responds to NO induction [85]. Based on biochemical data, the evidence of trHbO in protection from NO is weak, while it is more likely that this protein is involved in oxygen sensing and aerobic respiration [86]. Similar considerations could hold true for the enteric pathogen *C. jejuni*, which colonizes the intestinal tract of birds and can cause enteritis in humans. Like other pathogens, *C. jejuni* is exposed to NO and other nitrosating species during host infection [87], the expression of both bacterial Cj-trHbP and 3-on-3 globin being upregulated by NO [11]. The 3-on-3 globin plays the major role in resistance to nitrosative stress and aerobically converts NO to nitrate [88], whereas the contribution of trHbP is less prominent. In contrast, both globins are devoid of NO-protective activity under oxygen-limited conditions that normally exist *in vivo* [89]. Therefore, the role of Cj-trHbP is clearly distinct form that of the 3-on-3 globin, being related to O_2_ metabolism [7], [8], likely performing a peroxidase or P450-type of oxygen chemistry [9].

As a whole, the comparison of the biochemical properties of microbial globins, including trHbs, will allow to shed light on their functional diversification and on the molecular bases of microbe ecology.

## Supporting Information

File S1 **Supporting tables and figures.**
(DOCX)

## References

[1] Vinogradov SN, Hoogewijs D, Bailly X, Arredondo-Peter R, Guertin M, et al (2005) Three globin lineages belonging to two structural classes in genomes from the three kingdoms of life. Proc Natl Acad Sci USA 102: 11385–11389. 16061809 10.1073/pnas.0502103102 PMC1183549

[2] Vinogradov SN, Hoogewijs D, Bailly X, Mizuguchi K, Dewilde S, et al (2007) A model of globin evolution. Gene 398: 132–142. 17540514 10.1016/j.gene.2007.02.041

[3] Vuletich DA, Lecomte JT (2006) A phylogenetic and structural analysis of truncated hemoglobins. J Mol Evol 62: 196–210. 16474979 10.1007/s00239-005-0077-4

[4] Ascenzi P, Bolognesi M, Milani M, Guertin M, Visca P (2007) Mycobacterial truncated hemoglobins: from genes to functions. Gene 398: 42–51. 17532149 10.1016/j.gene.2007.02.043

[5] Pesce A, Bolognesi M, Nardini M (2013) The diversity of 2/2 (truncated) globins,. Adv Microb Physiol 63: 49–78. 24054794 10.1016/B978-0-12-407693-8.00002-9

[6] Vinogradov SN, Tinajero-Trejo M, Poole RK, Hoogewijs D (2013) Bacterial and archaeal globins - a revised perspective. Biochim Biophys Acta 1834: 1789–1800. 23541529 10.1016/j.bbapap.2013.03.021

[7] Wainwright LM, Elvers KT, Park SF, Poole RK (2005) A truncated haemoglobin implicated in oxygen metabolism by the microaerophilic food-borne pathogen *Campylobacter jejuni* . Microbiology 151: 4079–4091. 16339953 10.1099/mic.0.28266-0

[8] Wainwright LM, Wang Y, Park SF, Yeh SR, Poole RK (2006) Purification and spectroscopic characterization of Ctb, a group III truncated hemoglobin implicated in oxygen metabolism in the food-borne pathogen *Campylobacter jejuni* . Biochemistry 45: 6003–6011. 16681372 10.1021/bi052247k PMC2528550

[9] Lu C, Egawa T, Wainwright LM, Poole RK, Yeh SR (2007) Structural and functional properties of a truncated hemoglobin from a food-borne pathogen *Campylobacter jejuni* . J Biol Chem 282: 13627–13636. 17339325 10.1074/jbc.M609397200

[10] Gupta S, Pawaria S, Lu C, Hade MD, Singh C, et al (2012) An unconventional hexacoordinated flavohemoglobin from *Mycobacterium tuberculosis* . J Biol Chem 287: 16435–16446. 22437825 10.1074/jbc.M111.329920 PMC3351305

[11] Tinajero-Trejo M, Shepherd M (2013) The globins of *Campylobacter jejuni* . Adv Microb Physiol 63: 97–145. 24054796 10.1016/B978-0-12-407693-8.00004-2

[12] Ascenzi P, Visca P (2008) Scavenging of reactive nitrogen species by mycobacterial truncated hemoglobins. Methods Enzymol 436: 317–337. 18237641 10.1016/S0076-6879(08)36018-2

[13] Davidge KS, Dikshit KL (2013) Haemoglobins of Mycobacteria: structural features and biological functions. Adv Microb Physiol 63: 147–194. 24054797 10.1016/B978-0-12-407693-8.00005-4

[14] Ascenzi P, Coletta A, Cao Y, Trezza V, Leboffe L, et al (2013) Isoniazid inhibits the heme-based reactivity of *Mycobacterium tuberculosis* truncated hemoglobin N. Plos One 8: e69762. 23936350 10.1371/journal.pone.0069762 PMC3731299

[15] Arya S, Sethi D, Singh S, Hade MD, Singh V, et al (2013) Truncated hemoglobin, HbN, is post-translationally modified in *Mycobacterium tuberculosis* and modulates host-pathogen interactions during intracellular infection. J Biol Chem 288: 29987–29999. 23983123 10.1074/jbc.M113.507301 PMC3795296

[16] Ouellet H, Ouellet Y, Richard C, Labarre M, Wittenberg B, et al (2002) Truncated hemoglobin HbN protects *Mycobacterium bovis* from nitric oxide. Proc Natl Acad Sci USA 99: 5902–5907. 11959913 10.1073/pnas.092017799 PMC122874

[17] Pathania R, Navani NK, Gardner AM, Gardner PR, Dikshit KL (2002) Nitric oxide scavenging and detoxification by the *Mycobacterium tuberculosis* haemoglobin, HbN in *Escherichia coli* . Mol Microbiol 45: 1303–1314. 12207698 10.1046/j.1365-2958.2002.03095.x

[18] Ouellet H, Juszczak L, Dantsker D, Samuni U, Ouellet YH, et al (2003) Reactions of *Mycobacterium tuberculosis* truncated hemoglobin O with ligands reveal a novel ligand-inclusive hydrogen bond network. Biochemistry 42: 5764–5774. 12741834 10.1021/bi0270337

[19] Ouellet H, Ranguelova K, Labarre M, Wittenberg JB, Wittenberg BA, et al (2007) Reaction of *Mycobacterium tuberculosis* truncated hemoglobin O with hydrogen peroxide: evidence for peroxidatic activity and formation of protein-based radicals. J Biol Chem 282: 7491–7503. 17218317 10.1074/jbc.M609155200

[20] Couture M, Yeh S, Wittenberg BA, Wittenberg JB, Ouellet Y, et al (1999) A cooperative oxygen-binding hemoglobin from Mycobacterium tuberculosis. Proc Natl Acad Sci USA 96: 11223–11228. 10500158 10.1073/pnas.96.20.11223 PMC18015

[21] Mukai M, Savard PY, Ouellet H, Guertin M, Yeh SR (2002) Unique ligand-protein interactions in a new truncated hemoglobin from *Mycobacterium tuberculosis* . Biochemistry 41: 3897–3905. 11900532 10.1021/bi0156409

[22] Nardini M, Pesce A, Labarre M, Richard C, Bolli A, et al (2006) Structural determinants in the group III truncated hemoglobin from *Campylobacter jejuni* . J Biol Chem 281: 37803–37812. 17023416 10.1074/jbc.M607254200

[23] Antonini E, Brunori M (1971) Hemoglobin and Myoglobin in their Reactions with Ligands. Amsterdam: North-Holland Publishing Co.

[24] Doyle MP, Pickering RA, DeWeert TM, Hoekstra JW, Pater D (1981) Kinetics and mechanism of the oxidation of human deoxyhemoglobin by nitrites. J Biol Chem 256: 12393–12398. 7298665

[25] Huang Z, Shiva S, Kim-Shapiro DB, Patel RP, Ringwood LA, et al (2005) Enzymatic function of hemoglobin as a nitrite reductase that produces NO under allosteric control. J Clin Invest 115: 2099–2107. 16041407 10.1172/JCI24650 PMC1177999

[26] Huang KT, Keszler A, Patel N, Patel RP, Gladwin MT, et al (2005) The reaction between nitrite and deoxyhemoglobin: reassessment of reaction kinetics and stoichiometry. J Biol Chem 280: 31126–31131. 15837788 10.1074/jbc.M501496200

[27] Shiva S, Huang Z, Grubina R, Sun J, Ringwood LA, et al (2007) Deoxymyoglobin is a nitrite reductase that generates nitric oxide and regulates mitochondrial respiration. Circ Res 100: 654–661. 17293481 10.1161/01.RES.0000260171.52224.6b

[28] Grubina R, Basu S, Tiso M, Kim-Shapiro DB, Gladwin MT (2008) Nitrite reductase activity of hemoglobin S (sickle) provides insight into contributions of heme redox potential versus ligand affinity. J Biol Chem 283: 3628–3638. 18056715 10.1074/jbc.M705222200

[29] Petersen MG, Dewilde S, Fago A (2008) Reactions of ferrous neuroglobin and cytoglobin with nitrite under anaerobic conditions. J Inorg Biochem 102: 1777–1782. 18599123 10.1016/j.jinorgbio.2008.05.008

[30] Salhany JM (2008) Kinetics of reaction of nitrite with deoxy hemoglobin after rapid deoxygenation or predeoxygenation by dithionite measured in solution and bound to the cytoplasmic domain of band 3 (SLC4A1). Biochemistry 47: 6059–6072. 18465875 10.1021/bi8000819

[31] Sturms R, DiSpirito AA, Hargrove MS (2011) Plant and cyanobacterial hemoglobins reduce nitrite to nitric oxide under anoxic conditions. Biochemistry 50: 3873–3878. 21495624 10.1021/bi2004312

[32] Tiso M, Tejero J, Basu S, Azarov I, Wang X, et al (2011) Human neuroglobin functions as a redox-regulated nitrite reductase. J Biol Chem 286: 18277–18289. 21296891 10.1074/jbc.M110.159541 PMC3093900

[33] Li H, Hemann C, Abdelghany TM, El-Mahdy MA, Zweier JL (2012) Characterization of the mechanism and magnitude of cytoglobin-mediated nitrite reduction and nitric oxide generation under anaerobic conditions. J Biol Chem 287: 36623–36633. 22896706 10.1074/jbc.M112.342378 PMC3476328

[34] Tiso M, Tejero J, Kenney C, Frizzell S, Gladwin MT (2012) Nitrite reductase activity of nonsymbiotic hemoglobins from *Arabidopsis thaliana* . Biochemistry 51: 5285–5292. 22620259 10.1021/bi300570v PMC3857030

[35] Ascenzi P, Tundo GR, Fanali G, Coletta M, Fasano M (2013) Warfarin modulates the nitrite-reductase activity of ferrous human serum heme-albumin. J Biol Inorg Chem 18: 939–946. 24037275 10.1007/s00775-013-1040-2

[36] Hoshino M, Ozawa K, Seki H, Ford PC (1993) Photochemistry of nitric oxide adducts of water-soluble iron(III) porphyrin and ferrihemoproteins studied by nanosecond laser photolysis. J Am Chem Soc 115: 9568–9575.

[37] Hoshino M, Maeda M, Konishi R, Seki H, Ford PC (1996) Studies on the reaction mechanism for reductive nitrosylation of ferrihemoproteins in buffer solutions. J Am Chem Soc 118: 5702–5707.

[38] Boffi A, Sarti P, Amiconi G, Chiancone E (2002) The interplay between heme iron and protein sulfhydryls in the reaction of dimeric *Scapharca inaequivalvis* hemoglobin with nitric oxide. Biophys Chem 98: 209–216. 12128199 10.1016/s0301-4622(02)00094-7

[39] Herold S, Puppo A (2005) Kinetics and mechanistic studies of the reactions of metleghemoglobin, ferrylleghemoglobin, and nitrosylleghemoglobin with reactive nitrogen species. J Biol Inorg Chem 10: 946–957. 16267660 10.1007/s00775-005-0047-8

[40] Ascenzi P, Bocedi A, Antonini G, Bolognesi M, Fasano M (2007) Reductive nitrosylation and peroxynitrite-mediated oxidation of heme-hemopexin. FEBS J 274: 551–562. 17229156 10.1111/j.1742-4658.2006.05609.x

[41] Ascenzi P, di Masi A, Gullotta F, Mattu M, Ciaccio C, et al (2010) Reductive nitrosylation of ferric cyanide horse heart myoglobin is limited by cyanide dissociation. Biochem Biophys Res Commun 393: 196–200. 20116365 10.1016/j.bbrc.2010.01.092

[42] Ascenzi P, Cao Y, di Masi A, Gullotta F, De Sanctis G, et al (2010) Reductive nitrosylation of ferric human serum heme-albumin. FEBS J 277: 2474–2485. 20456498 10.1111/j.1742-4658.2010.07662.x

[43] Ascenzi P, Pesce A, Nardini M, Bolognesi M, Ciaccio C, et al (2013) Reductive nitrosylation of *Methanosarcina acetivorans* protoglobin: a comparative study. Biochem Biophys Res Commun 430: 1301–1305. 23261459 10.1016/j.bbrc.2012.11.122

[44] Bateman H (1910) Solution of a system of differential equations occurring in the theory of radioactive transformations. Proc Cambridge Phil Soc 15: 423–427.

[45] Milani M, Ouellet Y, Ouellet H, Guertin M, Boffi A, et al (2004) Cyanide binding to truncated hemoglobins: a crystallographic and kinetic study. Biochemistry 43: 5213–5221. 15122887 10.1021/bi049870+

[46] Bolli A, Ciaccio C, Coletta M, Nardini M, Bolognesi M, et al (2008) Ferrous *Campylobacter jejuni* truncated hemoglobin P displays an extremely high reactivity for cyanide - a comparative study. FEBS J 275: 633–645. 18190529 10.1111/j.1742-4658.2007.06223.x

[47] Cassoly R, Gibson Q (1975) Conformation, co-operativity and ligand binding in human hemoglobin. J Mol Biol 91: 301–313. 171411 10.1016/0022-2836(75)90382-4

[48] Moore EG, Gibson QH (1976) Cooperativity in the dissociation of nitric oxide from hemoglobin. J Biol Chem 251: 2788–2794. 1262343

[49] Rohlfs RJ, Olson JS, Gibson QH (1988) A comparison of the geminate recombination kinetics of several monomeric heme proteins. J Biol Chem 263: 1803–1813. 3338995

[50] Chiancone E, Gibson QH (1989) Ligand binding to the dimeric hemoglobin from *Scapharca inaequivalvis*, a hemoglobin with a novel mechanism for cooperativity. J Biol Chem 264: 21062–21065. 2592367

[51] Van Doorslaer S, Dewilde S, Kiger L, Nistor SV, Goovaerts E, et al (2003) Nitric oxide binding properties of neuroglobin. A characterization by EPR and flash photolysis. J Biol Chem 278: 4919–4925. 12480932 10.1074/jbc.M210617200

[52] Fasano M, Antonini G, Ascenzi P (2006) O_2_-Mediated oxidation of hemopexin–heme(II)-NO. Biochem Biophys Res Commun 345: 704–712. 16696943 10.1016/j.bbrc.2006.04.154

[53] Abbruzzetti S, Faggiano S, Spyrakis F, Bruno S, Mozzarelli A, et al (2011) Oxygen and nitric oxide rebinding kinetics in nonsymbiotic hemoglobin AHb1 from *Arabidopsis thaliana* . IUBMB Life 63: 1094–1100. 22034287 10.1002/iub.546

[54] Nicoletti FP, Howes BD, Fittipaldi M, Fanali G, Fasano M, et al (2008) Ibuprofen induces an allosteric conformational transition in the heme complex of human serum albumin with significant effects on heme ligation. J Am Chem Soc 130: 11677–11688. 18681435 10.1021/ja800966t

[55] Ascenzi P, Cao Y, Tundo GR, Coletta M, Fanali G, et al (2011) Ibuprofen and warfarin modulate allosterically ferrous human serum heme-albumin nitrosylation. Biochem Biophys Res Commun 411: 185–189. 21726535 10.1016/j.bbrc.2011.06.130

[56] Bocedi A, De Sanctis G, Ciaccio C, Tundo GR, di Masi A, et al (2013) Reciprocal allosteric modulation of carbon monoxide and warfarin binding to ferrous human serum heme-albumin. PLoS One 8: e58842. 23555601 10.1371/journal.pone.0058842 PMC3605432

[57] Perutz MF (1979) Regulation of oxygen affinity of hemoglobin: influence of structure of the globin on the heme iron. Annu Rev Biochem 48: 327–386. 382987 10.1146/annurev.bi.48.070179.001551

[58] Bolognesi M, Bordo D, Rizzi M, Tarricone C, Ascenzi P (1997) Nonvertebrate hemoglobins: structural bases for reactivity. Prog Biophys Mol Biol 68: 29–68. 9481144 10.1016/s0079-6107(97)00017-5

[59] Banci L, Bertini I, Huber JG, Spyroulias GA, Turano P (1999) Solution structure of reduced horse heart cytochrome *c* . J Biol Inorg Chem 4: 21–31. 10499099 10.1007/s007750050285

[60] Pesce A, Dewilde S, Nardini M, Moens L, Ascenzi P, et al (2003) Human brain neuroglobin structure reveals a distinct mode of controlling oxygen affinity. Structure 11: 1087–1095. 12962627 10.1016/s0969-2126(03)00166-7

[61] de Sanctis D, Dewilde S, Pesce A, Moens L, Ascenzi P, et al (2004) Crystal structure of cytoglobin: the fourth globin type discovered in man displays heme hexa-coordination. J Mol Biol 336: 917–927. 15095869 10.1016/j.jmb.2003.12.063

[62] Trent JT 3rd, Kundu S, Hoy JA, Hargrove MS (2004) Crystallographic analysis of *Synechocystis* cyanoglobin reveals the structural changes accompanying ligand binding in a hexacoordinate hemoglobin. J Mol Biol 341: 1097–1108. 15289104 10.1016/j.jmb.2004.05.070

[63] Vallone B, Nienhaus K, Brunori M, Nienhaus GU (2004) The structure of murine neuroglobin: novel pathways for ligand migration and binding. Proteins 56: 85–92. 15162488 10.1002/prot.20113

[64] Vallone B, Nienhaus K, Matthes A, Brunori M, Nienhaus GU (2004) The structure of carbonmonoxy neuroglobin reveals a heme-sliding mechanism for control of ligand affinity. Proc Natl Acad Sci USA 101: 17351–17356. 15548613 10.1073/pnas.0407633101 PMC536024

[65] Hoy JA, Hargrove MS (2008) The structure and function of plant hemoglobins. Plant Physiol Biochem 4: 371–379. 10.1016/j.plaphy.2007.12.016 18321722

[66] Shepherd M, Barynin V, Lu C, Bernhardt PV, Wu G, et al (2010) The single-domain globin from the pathogenic bacterium *Campylobacter jejuni*: novel D-helix conformation, proximal hydrogen bonding that influences ligand binding, and peroxidase-like redox properties. J Biol Chem 285: 12747–12754. 20164176 10.1074/jbc.M109.084509 PMC2857070

[67] Fanali G, di Masi A, Trezza V, Marino M, Fasano M, et al (2012) Human serum albumin: from bench to bedside. Mol Aspects Med 33: 209–290. 22230555 10.1016/j.mam.2011.12.002

[68] Ouellet YH, Daigle R, Lagüe P, Dantsker D, Milani M, et al (2008) Ligand binding to truncated hemoglobin N from *Mycobacterium tuberculosis* is strongly modulated by the interplay between the distal heme pocket residues and internal water. J Biol Chem 283: 27270–27278. 18676995 10.1074/jbc.M804215200 PMC2556007

[69] Yeh SR, Couture M, Ouellet Y, Guertin M, Rousseau DL (2000) A cooperative oxygen binding hemoglobin from *Mycobacterium tuberculosis*. Stabilization of heme ligands by a distal tyrosine residue. J Biol Chem 275: 1679–1684. 10636862 10.1074/jbc.275.3.1679

[70] Ouellet H, Milani M, LaBarre M, Bolognesi M, Couture M, et al (2007) The roles of Tyr(CD1) and Trp(G8) in *Mycobacterium tuberculosis* truncated hemoglobin O in ligand binding and on the heme distal site architecture. Biochemistry 46: 11440–11450. 17887774 10.1021/bi7010288

[71] Pesce A, Bolognesi M, Nardini M (2013) Protoglobin: structure and ligand-binding properties. Adv Microb Physiol 63: 79–96. 24054795 10.1016/B978-0-12-407693-8.00003-0

[72] Silkstone G, Kapetanaki SM, Husu I, Vos MH, Wilson MT (2012) Nitric oxide binding to the cardiolipin complex of ferric cytochrome C. Biochemistry 51: 6760–6766. 22803508 10.1021/bi300582u

[73] Paoli M, Anderson BF, Baker HM, Morgan WT, Smith A, et al (1999) Crystal structure of hemopexin reveals a novel high-affinity heme site formed between two beta-propeller domains. Nat Struct Biol 6: 926–931. 10504726 10.1038/13294

[74] Herold S, Fago A, Weber RE, Dewilde S, Moens L (2004) Reactivity studies of the Fe(III) and Fe(II)NO forms of human neuroglobin reveal a potential role against oxidative stress. J Biol Chem 279: 22841–22847. 15020597 10.1074/jbc.M313732200

[75] Perutz MF (1989) Myoglobin and haemoglobin: role of distal residues in reactions with haem ligands. Trends Biochem Sci 14: 42–44. 2650040 10.1016/0968-0004(89)90039-x

[76] Harutyunyan EH, Safonova TN, Kuranova IP, Popov AN, Teplyakov AV, et al (1996) The binding of carbon monoxide and nitric oxide to leghaemoglobin in comparison with other haemoglobins. J Mol Biol 264: 152–161. 8950274 10.1006/jmbi.1996.0630

[77] Brucker EA, Olson JS, Ikeda-Saito M, Phillips GN Jr (1998) Nitric oxide myoglobin: crystal structure and analysis of ligand geometry. Proteins 30: 352–356. 9533619

[78] Miele AE, Santanché S, Travaglini-Allocatelli C, Vallone B, Brunori M, et al (1999) Modulation of ligand binding in engineered human hemoglobin distal pocket. J Mol Biol 290: 515–524. 10390349 10.1006/jmbi.1999.2869

[79] Chan NL, Kavanaugh JS, Rogers PH, Arnone A (2004) Crystallographic analysis of the interaction of nitric oxide with quaternary-T human hemoglobin. Biochemistry 43: 118–132. 14705937 10.1021/bi030172j

[80] Beetlestone JG, Adeosun OS, Goddard JE, Kushimo JB, Ogunlesi MM, et al (1976) Reactivity difference between haemoglobins. Part XIX. J Chem Soc Dalton Trans 1251–1278.

[81] Royer WE Jr, Hendrickson WA, Chiancone E (1989) The 2.4-Å crystal structure of *Scapharca* dimeric hemoglobin: cooperativity based on directly communicating hemes at a novel subunit interface. J Biol Chem 264: 21052–21061. 2592366

[82] Hargrove MS, Barry JK, Brucker EA, Berry MB, Phillips GN Jr, et al (1997) Characterization of recombinant soybean leghemoglobin a and apolar distal histidine mutants. J Mol Biol 266: 1032–1042. 9086279 10.1006/jmbi.1996.0833

[83] Milani M, Pesce A, Ouellet Y, Ascenzi P, Guertin M, et al (2001) *Mycobacterium tuberculosis* hemoglobin N displays a protein tunnel suited for O_2_ diffusion to the heme. EMBO J 20: 3902–3909. 11483493 10.1093/emboj/20.15.3902 PMC149180

[84] Milani M, Savard PY, Ouellet H, Ascenzi P, Guertin M, et al (2003) A TyrCD1/TrpG8 hydrogen bond network and a TyrB10TyrCD1 covalent link shape the heme distal site of *Mycobacterium tuberculosis* hemoglobin O. Proc Natl Acad Sci USA 100: 5766–5771. 12719529 10.1073/pnas.1037676100 PMC156275

[85] Pawaria S, Lama A, Raje M, Dikshit KL (2008) Responses of *Mycobacterium tuberculosis* hemoglobin promoters to *in vitro* and *in vivo* growth conditions. Appl Environ Microbiol 74: 3512–3522. 18390674 10.1128/AEM.02663-07 PMC2423021

[86] Pathania R, Navani NK, Rajmohan G, Dikshit KL (2002) *Mycobacterium tuberculosis* hemoglobin HbO associates with membranes and stimulates cellular respiration of recombinant *Escherichia coli* . J Biol Chem 277: 5293–5302. 10.1074/jbc.M111478200 11796724

[87] Iovine NM, Pursnani S, Voldman A, Wasserman G, Blaser MJ, et al (2008) Reactive nitrogen species contribute to innate host defense against *Campylobacter jejuni* . Infect Immun 76: 986–993. 18174337 10.1128/IAI.01063-07 PMC2258852

[88] Elvers KT, Wu G, Gilberthorpe NJ, Poole RK, Park SF (2004) Role of an inducible single-domain hemoglobin in mediating resistance to nitric oxide and nitrosative stress in *Campylobacter jejuni* and *Campylobacter coli* . J Bacteriol 186: 5332–5341. 15292134 10.1128/JB.186.16.5332-5341.2004 PMC490904

[89] Avila-Ramirez C, Tinajero-Trejo M, Davidge KS, Monk CE, Kelly DJ, et al (2013) Do globins in microaerophilic *Campylobacter jejuni* confer nitrosative stress tolerance under oxygen limitation? Antioxid Redox Signal 18: 424–431. 22816769 10.1089/ars.2012.4750 PMC3526894

[90] Helbo S, Dewilde S, Williams DR, Berghmans H, Berenbrink M, et al (2012) Functional differentiation of myoglobin isoforms in hypoxia-tolerant carp indicates tissue-specific protective roles. Am J Physiol Regul Integr Comp Physiol 302: R693–R701. 22170621 10.1152/ajpregu.00501.2011

[91] Guex N, Peitsch MC (1997) SWISS-MODEL and the Swiss-PdbViewer: an environment for comparative protein modeling. Electrophoresis 18: 2714–2723. 9504803 10.1002/elps.1150181505

