# Supplementary material for: Nitrosylation Mechanisms of Mycobacterium tuberculosis and Campylobacter jejuni Truncated Hemoglobins N, O, and P
Source: PLoS One. 2014 Jul 22;9(7):e102811. doi: 10.1371/journal.pone.0102811 (PMC4106858; doi:10.1371/journal.pone.0102811)
Supplement: File S1 — Supporting tables and figures. (DOCX) [file pone.0102811.s001.docx]

**SUPPORTING INFORMATION**

**Nitrosylation mechanisms of *Mycobacterium tuberculosis* and *Campylobacter jejuni* truncated hemoglobins N, O, and P**

Paolo Ascenzi ^a,^*, Alessandra di Masi ^a,b^, Grazia R. Tundo ^c,d^, Alessandra Pesce ^e^, Paolo Visca ^a^, and Massimo Coletta ^c,d^

^a^ Interdepartmental Laboratory of Electron Microscopy, University Roma Tre,

Roma, Italy

^b^ Department of Sciences, University Roma Tre, Roma, Italy

^c^ Department of Clinical Sciences and Translational Medicine, University of Roma “Tor Vergata”, Roma, Italy

^d^ Interuniversity Consortium for the Research on the Chemistry of Metals in Biological Systems, Bari, Italy

^e^ Department of Physics, University of Genova, Genova, Italy

* Corresponding author: phone: +39-06-5733-3621; fax: +39-06-5733-6321.

E-mail: ascenzi@uniroma3.it

**Table S1.** Values of thermodynamic and kinetic parameters for reductive nitrosylation of Mt-trHbN(III), at 20.0 °C.

-----------------------------------------------------------------------------------------------------------------

pH *l*_on_ *l*_off_ *L* *l*_off_/*l*_on_ *b*

(M^-1^ s^-1^) (s^-1^) (M) (M) (s^-1^)

-----------------------------------------------------------------------------------------------------------------

8.4 2.2×10^5^ 2.3 1.8×10^-5^ 1.0×10^-5^ 1.1×10^-3^

8.7 1.6×10^5^ 1.8 1.9×10^-5^ 1.1×10^-5^ 1.4×10^-3^

8.8 1.9×10^5^ 2.3 9.3×10^-6^ 1.2×10^-5^ 1.6×10^-3^

9.0 1.4×10^5^ 1.6 1.6×10^-5^ 1.1×10^-5^ 2.5×10^-3^

9.1 1.1×10^5^ 1.5 9.6×10^-6^ 1.4×10^-5^ 2.9×10^-3^

9.4 2.3×10^5^ 1.9 1.3×10^-5^ 8.3×10^-6^ 5.0×10^-3^

-----------------------------------------------------------------------------------------------------------------

**Table S2.** Values of thermodynamic and kinetic parameters for reductive nitrosylation of Mt-trHbO(III), at 20.0 °C.

-----------------------------------------------------------------------------------------------------------------

pH *l*_on_ *l*_off_ *L* *l*_off_/*l*_on_ *b*

(M^-1^ s^-1^) (s^-1^) (M) (M) (s^-1^)

-----------------------------------------------------------------------------------------------------------------

8.4 1.1×10^4^ 1.6 1.1×10^-4^ 1.5×10^-4^ 1.0×10^-3^

8.7 8.5×10^3^ 1.3 2.1×10^-4^ 1.5×10^-4^ 1.3×10^-3^

8.8 1.6×10^4^ 1.9 9.8×10^-5^ 1.2×10^-4^ 1.8×10^-3^

9.0 9.2×10^3^ 2.1 1.9×10^-4^ 2.3×10^-4^ 2.9×10^-3^

9.1 2.1×10^4^ 2.4 1.8×10^-4^ 1.1×10^-4^ 3.6×10^-3^

9.4 9.8×10^3^ 1.7 1.2×10^-4^ 1.7×10^-4^ 6.2×10^-3^

-----------------------------------------------------------------------------------------------------------------

**Table S3.** Values of thermodynamic and kinetic parameters for reductive nitrosylation of Cj-trHbP(III), at 20.0 °C.

-----------------------------------------------------------------------------------------------------------------

pH *l*_on_ *l*_off_ *L* *l*_off_/*l*_on_ *b*

(M^-1^ s^-1^) (s^-1^) (M) (M) (s^-1^)

-----------------------------------------------------------------------------------------------------------------

8.4 1.7×10^5^ 8.3 5.3×10^-5^ 4.9×10^-5^ 2.8×10^-3^

8.7 9.1×10^4^ 5.9 4.9×10^-5^ 6.5×10^-5^ 4.8×10^-3^

8.8 1.6×10^5^ 6.5 4.6×10^-5^ 4.1×10^-5^ 7.0×10^-3^

9.0 1.1×10^5^ 8.1 6.5×10^-5^ 7.4×10^-5^ 1.1×10^-2^

9.1 8.9×10^4^ 6.7 7.9×10^-5^ 7.5×10^-5^ 1.3×10^-2^

9.4 1.6×10^5^ 7.1 3.1×10^-5^ 4.4×10^-5^ 2.3×10^-2^

-----------------------------------------------------------------------------------------------------------------

**Fig. S1.** Mt-trHbO(II) nitrosylation at 20.0 °C. (A) Difference absorbance spectrum of Mt-trHbO(II) *minus* Mt-trHbO(II)-NO, at pH 7.0. (B) Difference absorbance spectrum of Mt-trHbO(II) *minus* Mt-trHbO(II)-NO, at pH 9.0. (C) Normalized averaged time courses of Mt-trHbO(II) nitrosylation, at pH 7.0. The NO concentration was 4.0×10^-5^ M (trace a) and 1.2×10^-4^ M (trace b). The time course analysis according to Eqn. (1) allowed the determination of the following values of *k* = 9.3 s^-1^ (trace a) and 2.2×10^1^ s^-1^ (trace b). (D) Normalized averaged time courses of Mt-trHbO(II) nitrosylation, at pH 9.0. The NO concentration was 4.0×10^-5^ M (trace a) and 1.2×10^-4^ M (trace b). The time course analysis according to Eqn. (1) allowed the determination of the following values of *k* = 8.6 s^-1^ (trace a) and 3.0×10^1^ s^-1^ (trace b). (E) Dependence of the pseudo-first-order rate-constant *k* for Mt-trHbO(II) nitrosylation on the NO concentration, at pH 7.0. The analysis of data according to Eqn. (2) allowed the determination of *k*_on_ = (1.9±0.2)×10^5^ M^-1^ s^-1^. (F) Dependence of the pseudo-first-order rate-constant *k* for Mt-trHbO(II) nitrosylation on the NO concentration, at pH 9.0. The analysis of data according to Eqn. (2) allowed the determination of *k*_on_ = (2.3±0.3)×10^5^ M^-1^ s^-1^. The Mt-trHbO(II) concentration was 2.3×10^-6^ M. The NO concentration was 1.0×10^-4^ M (panels A and B). Where not shown, the standard deviation is smaller than the symbol. For details, see text.

**Fig. S2.** Cj-trHbP(II) nitrosylation at 20.0 °C. (A) Difference absorbance spectrum of Cj-trHbP(II) *minus* Cj-trHbP(II)-NO, at pH 7.0. (B) Difference absorbance spectrum of Mt-trHbP(II) *minus* Mt-trHbP(II)-NO, at pH 9.0. (C) Normalized averaged time courses of Cj-trHbP(II) nitrosylation, at pH 7.0. The NO concentration was 6.0×10^-6^ M (trace a) and 1.6×10^-5^ M (trace b). The time course analysis according to Eqn. (1) allowed the determination of the following values of *k* = 8.1×10^1^ s^-1^ (trace a) and 1.7×10^2^ s^-1^ (trace b). (D) Normalized averaged time courses of Cj-trHbP(II) nitrosylation, at pH 9.0. The NO concentration was 6.0×10^-6^ M (trace a) and 1.6×10^-5^ M (trace b). The time course analysis according to Eqn. (1) allowed the determination of the following values of *k* = 9.2×10^1^ s^-1^ (trace a) and 2.9×10^2^ s^-1^ (trace b). (E) Dependence of the pseudo-first-order rate-constant *k* for Cj-trHbP(II) nitrosylation on the NO concentration, at pH 7.0. The analysis of data according to Eqn. (2) allowed the determination of *k*_on_ = (1.1±0.2)×10^7^ M^-1^ s^-1^. (F) Dependence of the pseudo-first-order rate-constant *k* for Cj-trHbP(II) nitrosylation on the NO concentration, at pH 9.0. The analysis of data according to Eqn. (2) allowed the determination of *k*_on_ = (1.7±0.3)×10^7^ M^-1^ s^-1^. The Cj-trHbP(II) concentration was 1.6×10^-6^ M. The NO concentration was 1.0×10^-4^ M (panels A and B). Where not shown, the standard deviation is smaller than the symbol. For details, see text.

**Fig. S3.** Nitrite-mediated nitrosylation of Mt-trHbO(II), at 20.0 °C. (A) Difference absorbance spectrum of Mt-trHbO(II) *minus* Mt-trHbO(II)-NO, at pH 7.4. (B) Normalized averaged time courses of nitrite-mediated nitrosylation of Mt-trHbO(II), at pH 7.4. The nitrite concentration was 2.5×10^-3^ M (trace a) and 1.0×10^-2^ M (trace b). The time course analysis according to Eqn. (3) allowed the determination of the following values of *h* = 1.1×10^-3^ s^-1^ (trace a) and 3.8×10^-3^ s^-1^ (trace b). (C) Dependence of *h* on [NO_2_^-^] for nitrite-mediated nitrosylation of Mt-trHbO(II), at pH 7.4. The continuous line was generated from Eqn. (4) with *h*_on_ = (3.8±0.4)×10^-1^ M^-1^ s^-1^. (D) pH-Dependence of *h*_on_ for the nitrite-mediated nitrosylation of Mt-trHbO(II). The slope of the continuous line was -1.01±0.02 . The Mt-trHbO(II) concentration was 2.3×10^-6^ M. Where not shown, standard deviation is smaller than the symbol. For details, see text.

**Fig. S4.** Nitrite-mediated nitrosylation of Cj-trHbP(II), at 20.0 °C. (A) Difference absorbance spectrum of Cj-trHbP(II) *minus* Cj-trHbP(II)-NO, at pH 7.4. (B) Normalized averaged time courses of nitrite-mediated nitrosylation of Cj-trHbP(II), at pH 7.4. The nitrite concentration was 2.5×10^-3^ M (trace a) and 1.0×10^-2^ M (trace b). The time course analysis according to Eqn. (3) allowed the determination of the following values of *h* = 1.1×10^-2^ s^-1^ (trace a) and 4.4×10^-2^ s^-1^ (trace b). (C) Dependence of *h* on [NO_2_^-^] for nitrite-mediated nitrosylation of Cj-trHbP(II), at pH 7.4. The continuous line was generated from Eqn. (4) with *h*_on_ = 4.3±0.4 M^-1^ s^-1^. (D) pH-Dependence of *h*_on_ for the nitrite-mediated nitrosylation of Cj-trHbP(II). The slope of the continuous line was -1.00±0.03 . The Cj-trHbP(II) concentration was 1.6×10^-6^ M. Where not shown, standard deviation is smaller than the symbol. For details, see text.

**Fig. S5.** Mt-trHbO(III) reductive nitrosylation, at 20.0 °C. (A) Difference absorbance spectra of Mt-trHbO(III) *minus* Mt-trHbO(III)-NO and of Mt-trHbO(III)-NO *minus* Mt-trHbO(II)-NO (open and filled squares, respectively), at pH 9.0. (B) Normalized averaged time courses of Mt-trHbO(III) reductive nitrosylation, at pH 9.0. The NO concentration was 1.0×10^-4^ M (trace a) and 5.0×10^-4^ M (trace b). The time course analysis according to Eqns (5a)-(5c) allowed the determination of the following values of parameters α, *l*, and *b*: trace a - α = 0.33, *l* = 3.2 s^-1^, and *b* = 3.0×10^-3^ s^-1^; and trace b - α = 0.75, *l* = 6.6 s^-1^, and *b* = 2.9×10^-3^ s^-1^. (C) Dependence of *l* on [NO] for Mt-trHbO(III) reductive nitrosylation, at pH 9.0. The continuous line was generated from Eqn. (6) with *l*_on_ = (9.2±1)×10^3^ M^-1^ s^-1^ and *k*_off_ = 2.1±0.2 s^-1^. (D) Dependence of α on [NO] for Mt-trHbO(III) reductive nitrosylation, at pH 9.0. The continuous line was generated from Eqn. (7) with *L* = (1.9±0.2)×10^-4^ M. (E) Dependence of *b* on [NO] for Mt-trHbO(III) reductive nitrosylation, at pH 9.0. The average *b* value is 2.9×10^-3^ s^-1^ (dashed line). (F) Dependence of *b* on [OH^−^] for Mt-trHbO(III) reductive nitrosylation. The continuous line was generated from Eqn. (8) with *b*_OH−_ = (2.4±0.3)×10^2^ M^-1^ s^-1^ and *b*_H2O_ = (2.9±0.3)×10^-4^ s^-1^. The Mt-trHbO(III) concentration was 2.3×10^-6^ M. Where not shown, standard deviation is smaller than the symbol. For details, see text.

**Fig. S6.** Cj-trHbP(III) reductive nitrosylation, at 20.0 °C. (A) Difference absorbance spectra of Cj-trHbP(III) *minus* Cj-trHbP(III)-NO and of Cj-trHbP(III)-NO *minus* Cj-trHbP(II)-NO (open and filled triangles, respectively), at pH 9.0. (B) Normalized averaged time courses of Cj-trHbP(III) reductive nitrosylation, at pH 9.0. The NO concentration was 5.0×10^-5^ M (trace a) and 4.0×10^-4^ M (trace b). The time course analysis according to Eqns (5a)-(5c) allowed the determination of the following values of parameters α, *l*, and *b*: trace a - α = 0.47, *l* = 1.6×10^1^ s^-1^, and *b* = 9.8×10^-3^ s^-1^; and trace b - α = 0.90, *l* = 5.1×10^1^ s^-1^, and *b* = 1.2×10^-2^ s^-1^. (C) Dependence of *l* on [NO] for Cj-trHbP(III) reductive nitrosylation, at pH 9.0. The continuous line was generated from Eqn. (6) with *l*_on_ = (1.1±0.1)×10^5^ M^-1^ s^-1^ and *l*_off_ = 8.1±0.8 s^-1^. (D) Dependence of α on [NO] for Cj-trHbP(III) reductive nitrosylation, at pH 9.0 . The continuous line was generated from Eqn. (7) with *L* = (6.5±0.5)×10^-5^ M. (E) Dependence of *b* on [NO] for Cj-trHbP(III) reductive nitrosylation, at pH 9.0. The average *b* value is 1.1×10^-2^ s^-1^ (dashed line). (F) Dependence of *b* on [OH^−^] for Cj-trHbP(III) reductive nitrosylation. The continuous line was generated from Eqn. (8) with *b*_OH−_ = (9.1±1.0)×10^2^ M^-1^ s^-1^ and *b*_H2O_ = (4.8±0.5)×10^-4^ s^-1^. The Cj-trHbP(III) concentration was 1.6×10^-6^ M. Where not shown, standard deviation is smaller than the symbol. For details, see text.
